# 5-hydroxymethylcytosine marks promoters in colon that resist DNA hypermethylation in cancer

**DOI:** 10.1186/s13059-015-0605-5

**Published:** 2015-04-01

**Authors:** Santiago Uribe-Lewis, Rory Stark, Thomas Carroll, Mark J Dunning, Martin Bachman, Yoko Ito, Lovorka Stojic, Silvia Halim, Sarah L Vowler, Andy G Lynch, Benjamin Delatte, Eric J de Bony, Laurence Colin, Matthieu Defrance, Felix Krueger, Ana-Luisa Silva, Rogier ten Hoopen, Ashraf EK Ibrahim, François Fuks, Adele Murrell

**Affiliations:** Cancer Research UK Cambridge Institute, University of Cambridge, Robinson Way, Cambridge, CB2 0RE UK; Laboratory of Cancer Epigenetics, Université Libre de Bruxelles, Faculty of Medicine, Route de Lennik 808, 1070 Brussels, Belgium; Bioinformatics Group, The Babraham Institute, Babraham Research Campus, Cambridge, CB22 3AT UK; Department of Pathology, Addenbrooke’s Hospital, Box 231, Level 3, Hills Road, Cambridge, CB2 0QQ UK; Department of Biology and Biochemistry, Centre for Regenerative Medicine, University of Bath, Claverton Down, Bath, BA2 7AY UK

## Abstract

**Background:**

The discovery of cytosine hydroxymethylation (5hmC) as a mechanism that potentially controls DNA methylation changes typical of neoplasia prompted us to investigate its behaviour in colon cancer. 5hmC is globally reduced in proliferating cells such as colon tumours and the gut crypt progenitors, from which tumours can arise.

**Results:**

Here, we show that colorectal tumours and cancer cells express Ten-Eleven-Translocation (*TET*) transcripts at levels similar to normal tissues. Genome-wide analyses show that promoters marked by 5hmC in normal tissue, and those identified as TET2 targets in colorectal cancer cells, are resistant to methylation gain in cancer. *In vitro* studies of TET2 in cancer cells confirm that these promoters are resistant to methylation gain independently of sustained TET2 expression. We also find that a considerable number of the methylation gain-resistant promoters marked by 5hmC in normal colon overlap with those that are marked with poised bivalent histone modifications in embryonic stem cells.

**Conclusions:**

Together our results indicate that promoters that acquire 5hmC upon normal colon differentiation are innately resistant to neoplastic hypermethylation by mechanisms that do not require high levels of 5hmC in tumours. Our study highlights the potential of cytosine modifications as biomarkers of cancerous cell proliferation.

**Electronic supplementary material:**

The online version of this article (doi:10.1186/s13059-015-0605-5) contains supplementary material, which is available to authorized users.

## Background

Cancer is a complex disease characterised by genetic and epigenetic aberrations. DNA methylation, an epigenetic mark catalysed by *de novo* DNA methyltransferases (DNMT) [1], can modulate gene activity and its distribution across the genome is grossly disrupted in neoplasia [2]. The gain of methylation that frequently associates with the silencing of tumour suppressor genes can occur through the targeting of methylating complexes [3-5] but may also result from a failure to protect an unmethylated state [6]. Global losses, prominent across large expanses of the genome and thought to modulate genome function through higher order chromatin architectures [7-9], may occur through passive DNA demethylation caused by a failure to maintain DNA methylation during DNA replication [10]. The precise nature of the processes that govern DNA methylation changes in cancer are nevertheless still poorly defined and the recent discovery of active DNA demethylation mechanisms [11-16] bring about an additional level of complexity to our understanding of how such changes occur.

Methylation in DNA can be actively removed through oxidative demethylation by the TET family of alpha-glutarate-dependent oxygenases (TET1, TET2 and TET3) [15,17]. Further oxidation of 5hmC generates 5-formylcytosine (5fC) and 5-carboxylcytosine (5caC) [18] that are readily recognised by DNA repair processes [19]. The interconversion of cytosine modifications is now understood to be involved in the control of epigenetic plasticity and gene expression programmes [20].

Global reduction in 5hmC has been observed in all cancers studied to date [21-27], including colon cancer [21,28,29]. However, many but not all neoplasias show changes in expression levels of TETs [24-26,30-35]. Reduced levels of 5hmC in myelodysplasia and leukaemia frequently associate with mutations in TET2 [30,36,37] but changes in 5hmC levels are also thought to result from inhibition of TET activity by the onco-metabolite 2-hydroxyglutarate which accumulates through mutations of isocitrate dehydrogenases (IDH1/2) [38,39]. Importantly, reduced 5hmC does not always correlate with presence of IDH mutation [22,34] and IDH mutations are largely mutually exclusive to TET2 mutations in leukaemia [38]. In colon cancer on the other hand, mutations in TETs and IDHs are very rare [31,40]. Thus reduction of 5hmC appears to be a universal feature of tumourigenesis but factors implicated in regulating cytosine hydroxylation show tumour-type-specific aberrations.

The aim of this study was to provide an insight into the potential role of oxidative demethylation in the progressive changes in DNA methylation that occur in colon tumourigenesis. Molecular characterisation of the behaviour of 5hmC, 5mC and TETs in colon cancer tissues and cancer cells shows that changes in 5hmC levels in proliferating cells do not correlate with *TET* transcripts levels or with identifiable mutations in their catalytic domains. Importantly, we show that presence of 5hmC at promoters in normal tissues associates with resistance to methylation gain in colon cancer.

## Results and discussion

### Two distinct classes of 5hmC enrichment profiles are observed at active genes in normal human colon

**Figure 1:**
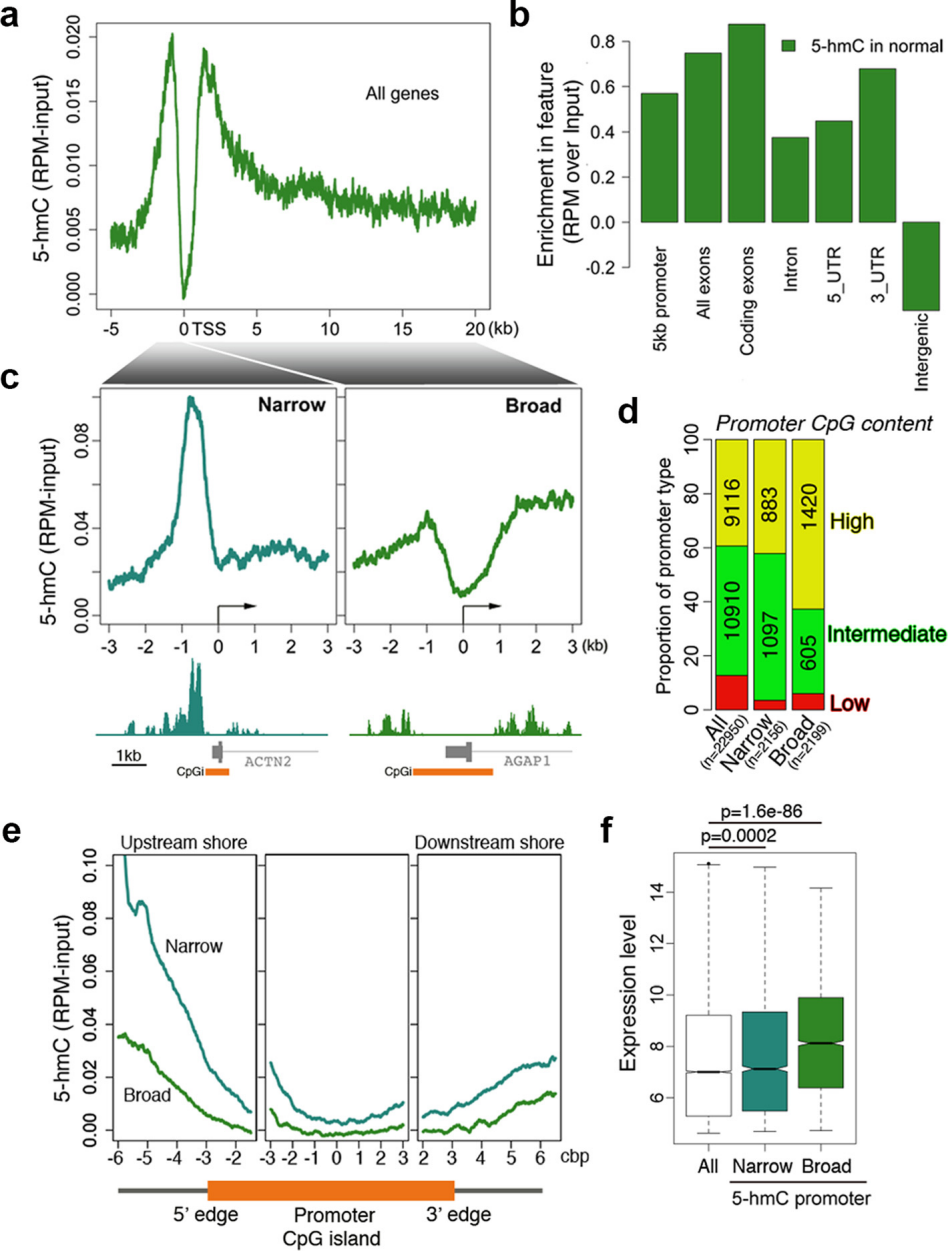
**5hmC promoter profiles and their association with active genes in normal colon. (a)** hmeDIP-seq profile for all genes around the TSS in normal colon tissue (n = 5). **(b)** Quantification of 5hmC enrichments in genomic features. **(c)** Two distinct promoter profiles were identified. Left panel: high 5hmC within a promoter window (-1 kb to +0.5 kb) with a ‘narrow’ promoter profile. Right panel: high 5hmC within gene bodies (from the TSS to the TTS) with a ‘broad’ promoter profile. Below are examples of each type of profile. **(d)** 5hmC and CpG content in the promoter. High, intermediate and low CpG content (HCP, ICP and LCP, respectively). Inset numbers represent the number of promoters for each category (LCP numbers not shown). **(e)** 5hmC content at promoter CpG islands. The levels represent an average of the population for each promoter type, thus individual loci may not necessarily display the full profile. Additional file 2 shows further examples. **(f)** Expression levels (log2 microarray intensity) of genes associated with 5hmC promoter profiles (*P* values were obtained by a Wilcox test).

We first set out to identify genes marked by 5hmC in colon by hmeDIP-seq in order to ultimately follow their methylation fate in cancer. Initial hmeDIP-seq on five DNA samples from normal mucosa of affected patients showed 5hmC enrichment at promoters, absent at the transcription start site (TSS), abundant within the body of genes and underrepresented within intergenic regions (Figure 1a and b).

From the profiling of 5hmC content across genes we identified two types of enrichments at gene promoters (Figure 1c). A ‘narrow’ type was observed after ranking 5hmC read content inside a window of -1 kb to +0.5 kb of the TSS and a ‘broad’ type after ranking by 5hmC read content in the gene body (from TSS to the TTS). We identified 2,156 unique ‘narrow’ and 2,199 unique ‘broad’ promoters (listed in Additional file 1).

The ‘narrow’ and ‘broad’ profiles were distinct in terms of promoter CpG content (Figure 1d) and in distribution of 5hmC around promoter CpG islands (Figure 1e). Promoters with the ‘narrow’ profile were enriched for intermediate CpG content promoters (ICP) whereas the ‘broad’ promoters where mostly high in CpG content (Figure 1d). Both promoter types showed that 5hmC is enriched within the shores of promoter CpG islands, more so within the upstream shore, and a higher overall content of 5hmC for the ‘narrow’ type (Figure 1e). Note, however, that the enrichment of 5hmC in the downstream shore of *ACTN2* is lower than that for *AGAP1*. The levels measured over the islands represent an average of the population for each type of promoter, and thus individual loci may not necessarily display the full enrichment profile across the associated promoter CpG island. Additional file 2 shows further examples to illustrate this. Interestingly, comparison of the 5hmC profiles with Illumina expression array data from four normal cases showed that ‘narrow’ promoter genes are less active than the ‘broad’ type (Figure 1f), in accordance with previous correlations made for higher 5hmC content at promoters and reduced gene activity in mouse and human ES cells [41,42]. Biological processes also typified the 5hmC promoters; gene ontology categories indicative of gut function were enriched for the ‘narrow’ type whereas cell differentiation and development where enriched for the ‘broad’ type (Additional file 2).

Together these data show that the content and distribution of 5hmC within promoters and gene bodies correlates with gene activities involved in normal gut epithelial function and differentiation.

### 5hmC enrichment is similar to 5mC enrichment at genic regions

Next we examined DNA methylation content with respect to the 5hmC profiles by comparing our hmeDIP-seq data to published meDIP-seq data for normal colon tissue [43] (Additional file 3). We generated heatmaps for 5hmC and 5mC enrichment profiles from -3 kb to +20 kb around the TSS (Additional file 3a). Ten clusters were generated based on the distribution of 5hmC and 5mC within this window. Overall we found that where 5hmC-specific enrichment is observed, the enrichment profiles are similar for 5mC (Additional file 3a). The exception was cluster 2 where there was more DNA methylation near the TSS than 5 hmC. Further comparison of 5hmC and 5mC profiles closer to the TSS (-3 kb to +3 kb) of all loci suggest that the differences in enrichment patterns for 5hmC and 5mC occur near the TSS and upstream promoter region (Additional file 3b). This suggests that several gene promoters may have DNA methylation without 5 hmC.

The heatmaps also identified the ‘narrow’ promoters as typified by clusters 3 and 8 whereas the ‘broad’ promoters fell within clusters 5, 6, 7 and 9 (Additional file 3c). With the exception of clusters 2 and 3 that showed an enrichment for LCP promoters, most of the 5hmC/5mC clusters fell with promoters of an intermediate or high CpG content (Additional file 3e).

We then compared the meDIP-seq methylation clusters to the methylation levels assessed by the Infinium27k arrays in 17 normal samples from our patient cohort (Additional file 3e). For the loci plotted in the heatmap the maximal distance of the Infinium probes to the TSS is 1499 bp. The highest methylation levels for these probes were around the promoters grouped within clusters 1 and 2, which correspond to the meDIP-seq data where the highest methylation enrichment was observed (Additional file 3a and e). Similarly clusters 4 to 9 which all reported low amounts of DNA methylation around the TSS by meDIP-seq also had lower levels of DNA methylation at the corresponding Infinium probes (Additional file 3a and e).

Thus in our normal colon tissues, the Infinium arrays concur with meDIP-seq enrichment patterns proximal to the TSS of genes.

### Reduced levels of 5hmC in colon tumours do not correlate with changes in TET transcript levels

**Figure 2:**
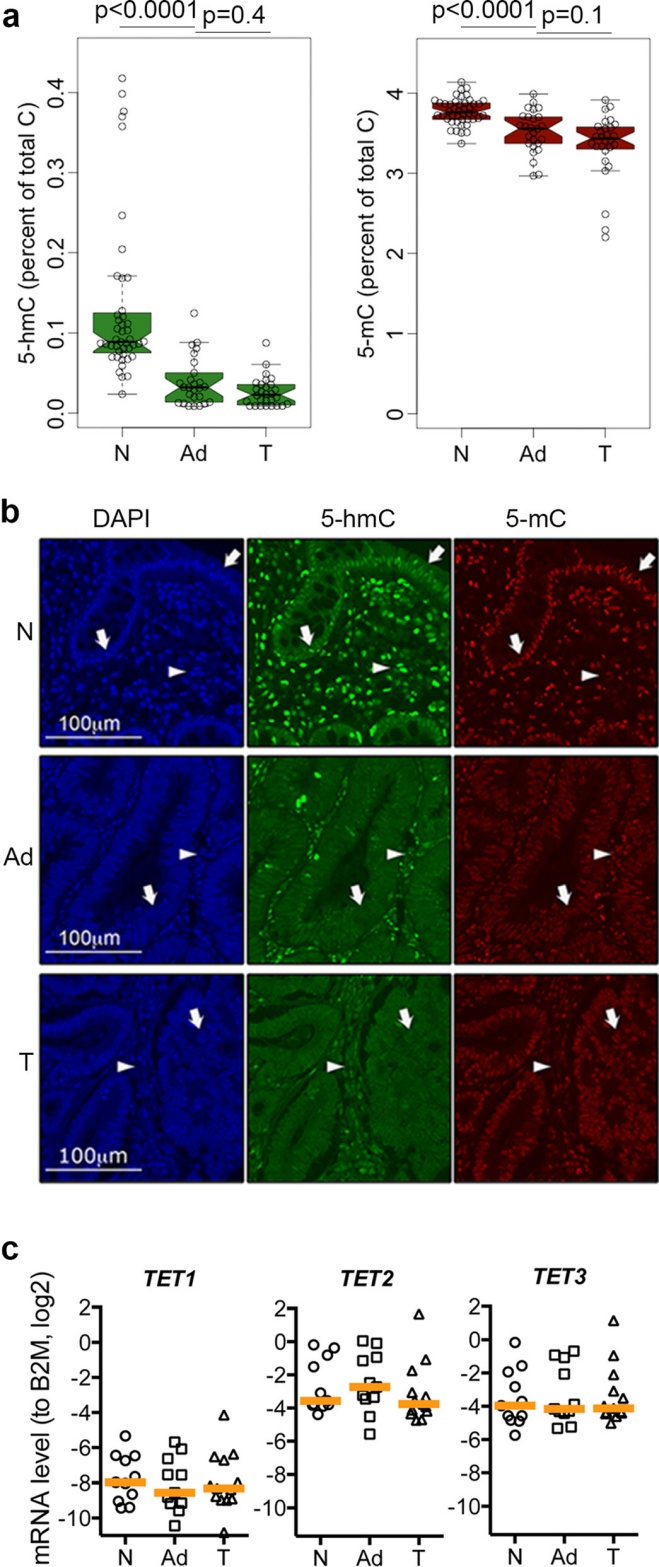
**Reduced 5hmC in tumours without global changes in** ***TET*** **s transcripts. (a)** Global content of 5hmC and 5mC in normal (N), adenoma (Ad) and adenocarcinoma (T) DNA by mass spectrometry (*P* values were obtained by a Wilcox test). **(b)** Representative images from a colon cancer tissue microarray immunofluorescence. Arrows indicate the epithelium, arrowheads the stroma. **(c)** Absolute levels of *TET*s (standard curve method) in selected cases from our colon cancer cohort. Orange vertical bands represent the median. Negative values indicate *TET*s transcripts are less abundant than *B2M* transcripts. There was no significant change in levels across tissues but considerable variation within tissues.

Having established profiles for 5hmC and 5mC in normal colon we next analysed their behaviour in neoplasia. Our colon cancer cohort is composed of 47 normal tissues, 36 adenomas and 31 adenocarcinomas (Additional file 1). We confirmed that 5hmC and 5mC are globally reduced during colon cancer progression using liquid chromatography mass spectrometry (LCMS) and immunofluorescence (IF) (Figure 2a and b). The IF also shows that 5hmC is concentrated in the differentiated colon epithelium and is low in the base of the crypts and tumours consistent with previous reports [21]. Importantly, we observed *TET1*, *TET2* and *TET3* were consistently transcribed in normal and tumour tissue and that the absolute levels of *TET1* were low relative to *TET2* and *TET3* by Sybr-Green qRT-PCR (Figure 2c). Further analysis of *TET* expression in normal-tumour matched cases by Taqman qRT-PCR showed no correlation with the changes in global levels of 5hmC (Additional file 4). Moreover, mining of recently published data sets [31,44] indicates that *TET*s are present in normal crypt and differentiated epithelium and tumours.

Mutation at the Fe2 and a-KG binding pockets could account for a lack of TET activity [30] but these were specifically excluded in our sample set through targeted exonic sequencing (Additional file 5a and Additional methods). We identified non-synonymous mutations elsewhere in the catalytic domains of *TET*s but their presence did not correlate with the changes in global 5hmC levels (Additional file 5b). Reduction of 5hmC in tumours may also be due to inhibition of TETs by metabolites that accumulate through mutation of IDH1/2, Fumarate hydratase (FH) or Succinate dehydrogenase (SDH) [39,45]. In our study IDH1/2 mutations were excluded in a subset of samples (not shown) and recent larger studies have shown IDH1/2, FH or SDH mutation is rare or absent in colon cancer [31,40].

We do not have TET protein data associated with our sample set and therefore we cannot exclude that the global reduction in 5hmC could be due to post-transcriptional events with an impact on variations in the stability or activity of TETs. However, the detection of mRNA at levels similar to the normal tissues suggests that the reduced levels of 5hmC that we uncover in all our colon tumours is unlikely to be due to an absence or mutation of TETs or an inhibition by currently recognised onco-metabolites.

### 5hmC is reduced across the genome of tumours with a small effect on gene transcription

We profiled 5hmC in four matching adenocarcinomas. The hmeDIP-seq read content in tumours showed an overall similar distribution to the normal tissue but with markedly reduced 5hmC levels across the genome as assessed by 5hmC content within repetitive elements (Additional file 6) and within genes (Additional file 7a and b). The reduced level of 5hmC in tumours compared to normal was confirmed at selected loci by a glycosylase-restriction enzyme sensitive assay (gluc-MS-qPCR - Additional file 7c) indicating that genes continue to be marked by a reduced amount of 5hmC in tumours.

Illumina expression array data generated from four normals and 14 tumours showed a small but statistically significant reduction in gene activity for genes with ‘broad’ 5hmC promoters (Additional file 7d). Thus, although 5hmC associates with active gene transcription, the reduction of 5hmC in tumours were accompanied by very small expression level changes. These results indicate that genes that acquire 5hmC in normal colon are transcriptionally active in tumours and suggest that low levels of 5hmC do not hinder transcription.

### Loci marked by 5hmC in normal have an innate resistance to DNA hypermethylation in cancer

To ascertain whether promoters normally marked by 5hmC undergo DNA methylation changes in colon cancer, we assessed DNA methylation in 17 tumours matched to the normal tissues using Infinium methylation arrays. The Infinium27k arrays are a robust platform for quantitative measurement of the DNA methylation status of 27,578 CpG sites located at the promoter regions of 14,495 protein-coding genes [43,46]. Infinium technology is based on bisulfite conversion that does not distinguish between 5mC and 5hmC. However, 5hmC only makes up a small percentage of modified cytosines in normal colon and an even smaller percentage in colon cancer tissue. Based on the median levels of 5hmC detected by LCMS (Figure 2), only about 2.4% of 5mC reported in the Infinium data is likely to be undistinguishable from 5hmC in normal cells, and about 0.7% in tumours.

**Figure 3:**
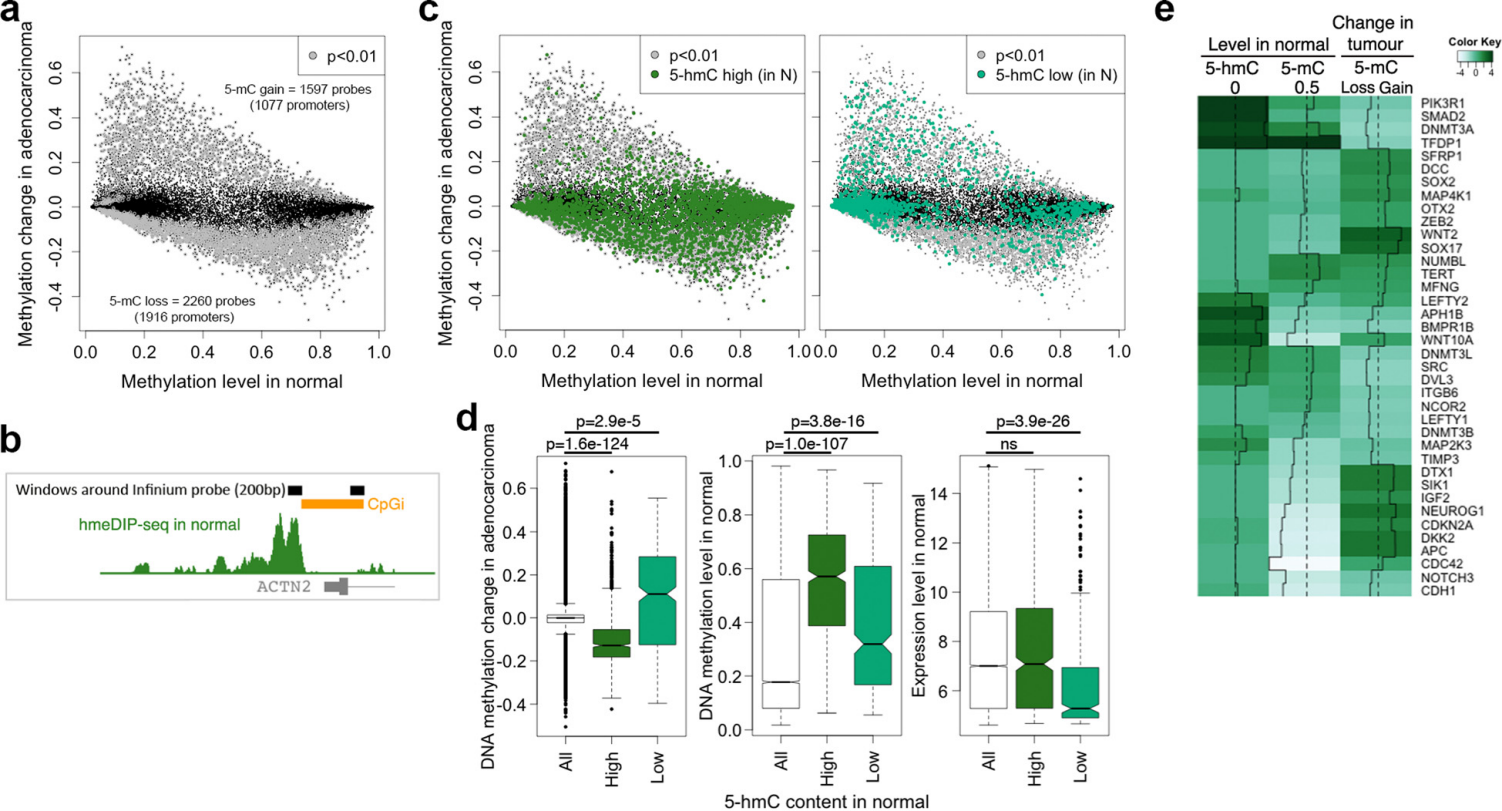
**Promoters marked by 5hmC in normal colon resist DNA methylation gain in tumours. (a)** DNA methylation changes in adenocarcinoma (n = 17) relative to matched normal tissues (n = 17) (Infinium arrays). Each dot represents a CpG (grey dots are changes with *P* <0.01). **(b)** 5hmC read content measured in windows around the Infinium probes (black bars). CpG island (CpGi) as orange bar. **(c)** Overlay of 5hmC high or 5hmC low promoters on the methylation states. **(d)** Left panel: 5hmC content around the Infinium probes of promoters with a significant change in methylation. High 5hmC promoters are prone to loss of DNA methylation in tumours whereas low 5hmC promoters are prone to methylation gain in tumours (limma geneSetTest). Middle panel: 5hmC content in normal and levels of DNA methylation in normal to show that methylation gain or loss occurs across a range of methylation levels in normal (*P* values from a Wilcox test). Right panel: 5hmC content in normal and expression levels in normal. DNA methylation prone genes (5hmC low) have low expression in the normal tissue (*P* values from a Wilcox test). **(e)** Heatmap comparing 5hmC and 5mC levels in normal to the 5mC changes in tumours at selected loci.

Methylation changes in our patient cohort showed both gain and loss of promoter DNA methylation (Figure 3a). To refine our analysis of 5hmC content to changes in DNA methylation at the promoters assessed by the Infinium platform, we counted the hmeDIP-seq reads from normals in 200 bp windows around the Infinium probes (Figure 3b). After ranking by read content we identified the top 3,000 5hmC enriched loci (5hmC-high) as well as 3,000 loci where 5hmC was low or undetected (5hmC-low). Interestingly, by this measurement of read counts around the Infinium probes, we observed that promoters with high 5hmC in normal are either resistant to methylation change or are prone to methylation loss (79% loss vs. 21% gain from 676 probes with significant change out of 3,000) and that 5hmC marked promoters more frequently associate with a range of intermediate levels of methylation in normal (Figure 3c left panel and d). 5hmC low promoters more frequently associated with low levels of methylation in normal and showed an increased propensity to methylation gain, albeit methylation loss was also observed (56% gain vs. 44% loss from 379 probes with significant change out of 3,000) (Figure 3c right panel and d). We also find that the methylation-prone genes that lack 5hmC in normal have a low level of expression in the normal tissue (Figure 3d, right panel), in agreement with a recent report where propensity to methylation gain in tumours is frequent at promoters of genes with low expression in the normal tissue [47].

Importantly, the reciprocal pattern of high/low 5hmC in normal with loss/gain of methylation in adenocarcinoma was already present at the adenoma stages (Additional file 8a and b) and observed at CpG islands and island shores (Additional file 8c). This reciprocal pattern was also present at previously identified colon cancer-specific small regions of differential DNA methylation (sDMRs) [8] (Additional file 8d) and clearly observed and verified in a number of colon cancer relevant gene promoters (Figure 3e and Additional file 9).

Together these results indicate that gene promoters marked with 5hmC in normal rarely become hypermethylated when 5hmC is reduced in tumours. Indeed these promoters have a tendency to lose DNA methylation in cancer. We also identified 117 promoters where 5hmC was still detected in adenocarcinomas, albeit at very low levels, and found that these where three times more likely to have lost methylation rather than gain (27% vs. 8.5%, respectively) (Additional file 10). These results may suggest that DNA demethylation at a subset of proximal promoters could be mediated via hydroxymethylation and/or that the presence of 5hmC helps to repel DNA methylating complexes as previously suggested [48,49].

There is strong evidence from cell labelling experiments that colon cancer can originate from the stem cell/progenitor compartment [50]. Our data, and that of others [21], showing that global 5hmC levels are low in the stem cell compartment and in cancer tissues may suggest that 5hmC is not lost in colon cancer. Rather, 5hmC may not accumulate due to an aberrant progenitor-like proliferative state. One explanation for why the loci that would accumulate 5hmC upon terminal differentiation are seemingly more resistant to gain of DNA methylation in cancer, in contrast with loci that do not accumulate 5hmC, could be that the TETs in cancer cells are bound to their target promoters to prevent *de novo* DNA methylation.

### TET2 marks promoters in cancer cells that resist DNA methylation gain in primary tumours but is not required to maintain a demethylated state

In order to examine whether TETs are bound to DNA in cancer cells we turned to the colorectal cancer cell line HCT116. This cell line shows low global levels of 5hmC and *TET2* and *TET3* transcript levels comparable to that observed in normal and adenocarcinoma tissue (Additional file 11a to c). Despite the extremely low global content of 5hmC in these cells, lower than that seen in the primary tissues, TET2 and TET3 proteins can be detected in the nuclear fraction (Additional file 11d) albeit a sizeable amount of TET2 is present in the cytoplasm (Additional file 11d and e). A similar subcellular distribution of TET2 is observed in normal colon crypts and tumours by immunohistochemistry (Additional file 12).

**Figure 4:**
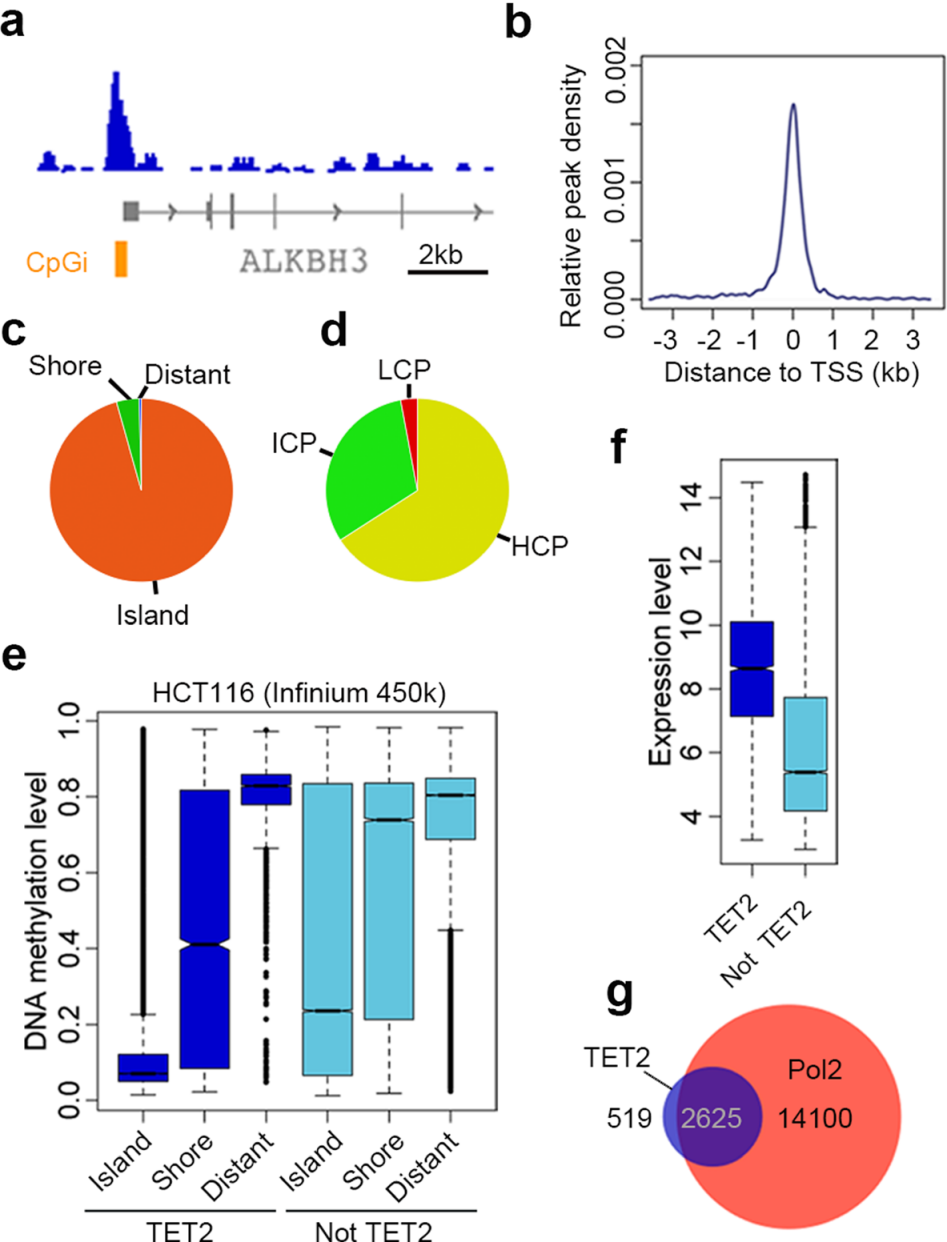
**TET2 binds promoters of active genes in cancer cells. (a)** Example of TET2 binding profile in HCT116 colorectal cancer cells. **(b)** TET2 binds close to TSSs and **(c, d)** primarily at CpG islands within HCP promoters. **(e)** TET2 bound islands are largely unmethylated and **(f, g)** associate with active genes.

Chromatin immunoprecipitation sequencing (ChIP-seq) revealed that TET2 preferentially binds to gene promoters within 1 kb of the TSS (Figure 4a and b). Overall 3,144 promoters were identified as TET2 targets (Additional file 1) of which the large majority were CpG island-containing promoters of the HCP type (Figure 4c and d). CpG islands bound by TET2 were largely unmethylated as measured by Infinium450k arrays (from GSE29290) and CpG island shores showed lower methylation levels at the TET2 bound sites relative to those not bound by TET2 (Figure 4e). We validated a number of loci identified in the TET2 ChIP-seq by ChIP-qPCR (Additional file 13). Interestingly, presence of TET2 associated with active genes measured by expression arrays (GSE36133) or evidenced by a considerable overlap with RNA Pol2 binding sites (ENCODE Pol2 ChIP-seq) (Figure 4f and g).

**Figure 5:**
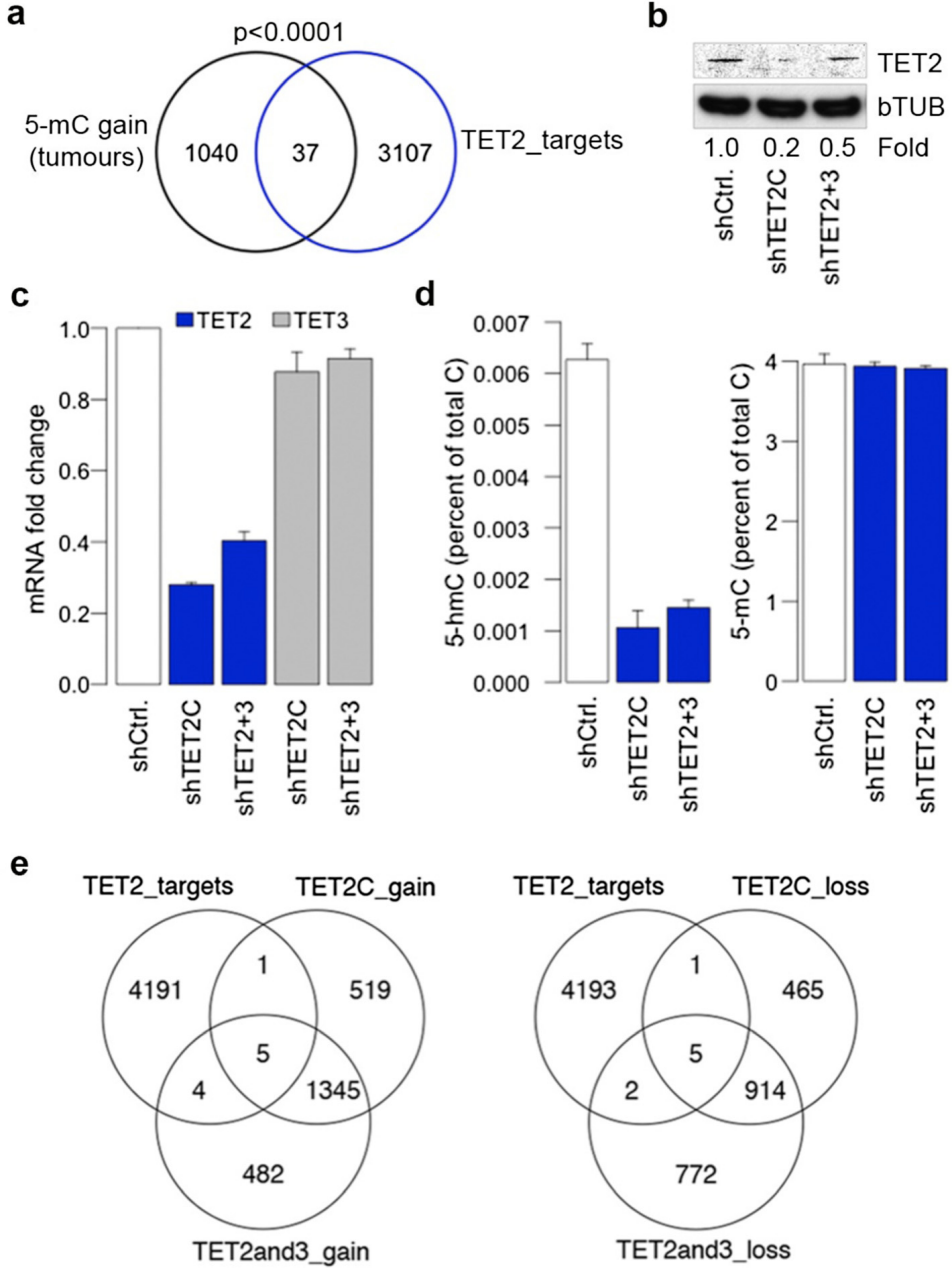
**Pervasive maintenance of a methylation-free state at TET2 bound promoters. (a)** DNA methylation gain in primary tumours was remarkably scarce at the TET2 bound promoters identified in HCT116 cells (*P* <0.0001, binomial test). **(b)** Western blot for TET2 and beta TUBULIN from whole cell extracts of HCT116 cells stably transfected with a non-targeting shRNA control (shCtrl.) or with shRNA to TET2 (TET2C) or to TET2 and TET3 (TET2 + 3). Fold change in the knockdown was calculated relative to the shCtrl. **(c)** qRT-PCR for *TET2* and *TET3*. **(d)** Global levels of 5hmC and 5mC by LCMS. **(e)** DNA methylation changes by Infinium arrays after depletion of TET2.

If the TETs bind to DNA and protect against hypermethylation in tumours, then it would be expected that promoters susceptible to DNA methylation gain in colon tumours would form a distinct group with a minimal overlap with TET target promoters. We therefore examined whether loci that gained DNA methylation in our primary tumours (1,597 probes for 1,077 promoters) were likely TET2 target promoters (4,201 probes for 3,144 promoters). This analysis showed less than 1% overlap between loci that gain DNA methylation in tumours and the TET2 bound promoters (Figure 5a). These results could suggest that TET2 might be part of a mechanism that protects promoters from *de novo* DNA methylation. To examine this we depleted TET2 in HCT116 cells by stable transfection of shRNAs (Figure 5b and c). In one instance we used shRNA against TET2 alone (TET2C) and in the other shRNA against TET2 and TET3 (TET2 + 3 where TET3 mRNA was not affected and therefore treat this sample as a TET2 only knockdown) (Figure 5c). LCMS after TET2 depletion showed a marked reduction in the global level of 5hmC (Figure 5d), confirming TET2 oxygenase activity in HCT116, without changes in global levels of 5mC (Figure 5d) but this could be due to the small contribution of promoter methylation to the methylome. Infinium arrays identified several loci with changes in DNA methylation (Figure 5e) that were for the most part low in magnitude (median of change was 10.4%; not shown). Similar changes in levels of DNA methylation were recently observed after TET1 depletion in differentiated cells [51]. However in our study, methylation levels at TET2 bound CpG islands were largely unaffected after TET2 depletion (less than 1%, Figure 5e), suggesting that these promoters do not require high levels of TET2 to maintain the methylation free state and are intrinsically resistant to methylation changes.

Survival outcomes estimated from publicly available colorectal cancer datasets [52,53] further indicate that *TET2* expression levels do not significantly associate with patient survival, which is consistent with the small effect that we see in these *in vitro* TET2 studies. TET2 therefore seems to play a moderate role in controlling cytosine modifications during gut tumourigenesis.

### Promoters with high levels of 5hmC in normal colon overlap with bivalently marked promoters in human embryonic stem cells that do not become methylated in colon cancer

If tumours arise from intestinal cells in the crypt and if 5hmC is a mark of terminally differentiated cells, then how do we explain the resistance of 5hmC promoters to methylation gain in tumours prior to their accumulating 5hmC in normal tissue? TET2 depletion only has a moderate effect on DNA methylation in cancer cells, suggesting that the protective mechanism is unlikely to be due to continuous TET2 binding at target promoters. Although TET2 may not be involved in maintaining the unmethylated state of its target promoters, we cannot exclude that other proteins within a TET-complex may be involved. However there may be alternative explanations, one of which is that 5hmC promoters are epigenetically marked during early development to make them intrinsically unlikely to develop characteristics such as H3K27me3 in the soma that predispose to DNA methylation gain.

Precedents for early epigenetic marking include genomic imprinting and X-inactivation, but may also include the recently described instructive process for gain of methylation in cancer which occurs at promoters containing histone H3K4 and H3K27 tri-methylation (so-called bivalent promoters) in human embryonic stem cells (hESC) [54-58]. ESCs unlike most other proliferating cells already have high levels of 5hmC. In mouse ESCs Tet1 is found either at the TSS of bivalent promoters together with silencing complexes independent of 5hmC or downstream of the TSS together with 5hmC and the PRC2 complex [59,60]. In human ESC 5hmC has been found more at active gene promoters and enhancers than at poised (bivalent) enhancers [61].

**Figure 6:**
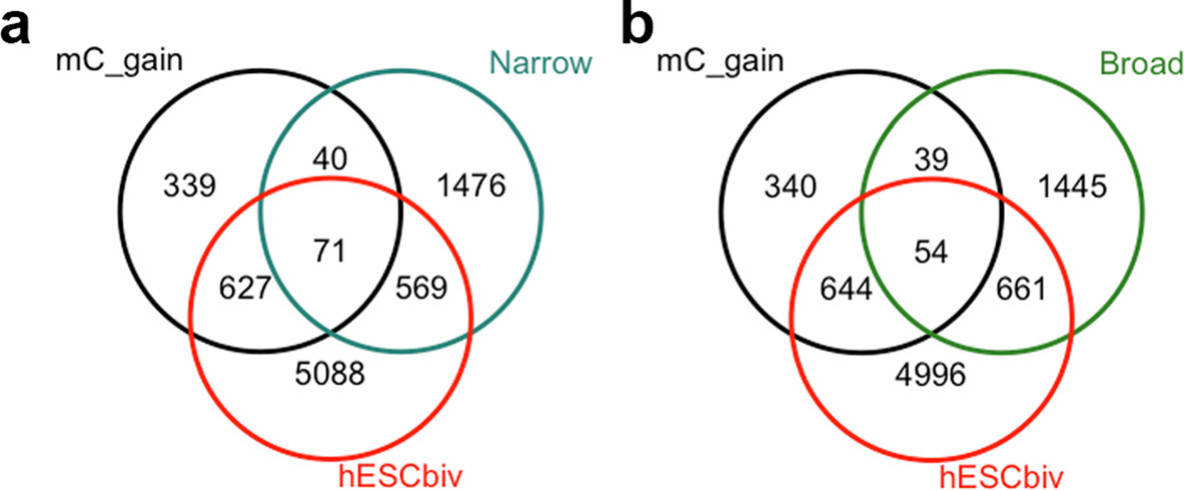
**5hmC marked promoters are not subject to histone-bivalency-mediated methylation gain.** Venn diagrams to illustrate a high incidence of promoter methylation gain in our cohort at promoters with H3K4me3/K27me3 bivalency in human embryonic stem cells (hESCbiv). The incidence of methylation gain is low at hESCbiv promoters marked by 5hmC in normal colon. **(a)** For narrow and **(b)** broad 5hmC promoters.

A comparison of our dataset of 5hmC marked promoters to a published dataset of hESC bivalent promoters [57] confirmed that approximately 65% of promoters that gain methylation in our colon cancer cohort are also bivalently marked in hESC (Figure 6a and b). Consequently we also examined the extent to which promoters marked by 5hmC in normal colon overlap with bivalently marked promoters in hESCs. We found that 30% of all 5hmC promoters overlapped with bivalent genes in hESCs (Figure 6a and b). Interestingly, these mostly coincided with bivalent promoters that do not become hypermethylated in colorectal cancer. This observation indicates that bivalent promoters can be broadly separated into discrete instructive categories: one for silencing after tissue differentiation and susceptible to methylation gain in cancer; and another for poised activation and acquisition of 5hmC with resistance to methylation gain in cancer. If 5hmC is acquired as an end point of instructive activation, this would fit with our data where we see 5hmC accumulating in terminally differentiated cells at genes that are active in both cancer and normal tissue.

## Conclusions

DNA methylation change is a prominent feature of cancer and in recent years, low levels of 5hmC have been reported as a hallmark of several cancers. We confirm that 5hmC is strongly reduced in colon cancer cells relative to the normal tissues. However, we also find that the *TET*s are present in cancer tissues, albeit at the transcript level, and with no evidence for mutations that could account for the decreased levels of 5hmC. We have recently shown that there is a delay in the generation of 5hmC on newly synthesised DNA [62] that can be responsible for the low levels of 5hmC in proliferating cells in the presence of TETs.

Genome-wide mapping shows that gene promoters marked by 5hmC seem distinctly resistant to DNA methylation gain, and slightly prone to DNA methylation loss. One explanation for this finding may be that the TETs continue to maintain the DNA methylation levels at promoters in the proliferating tumour cells. However, the marginal changes in DNA methylation at a significant number of gene promoters induced by the TET2 knockdowns were hardly observed at promoters we have identified as TET2 targets.

Correlative observations indicate that promoters with 5hmC in normal colon have a substantial overlap with bivalent promoters in hESCs, and may suggest that 5hmC is a mark of loci that have undergone a counteractive process that prevents the acquisition of hypermethylation-predisposing characteristics. Although we have potentially eliminated a maintenance role for TET2 in keeping the target promoters free of DNA methylation in colon cancer, this does not preclude the TETs from having an initiating function that marks genes for activation during early development. This initiating process could counter the instructive hypermethylation in cancer process by active removal of methylated DNA [19], by inhibiting *de novo* DNA methylation [48] and/or by attracting regulatory complexes to chromatin [63].

Finally, it has been suggested that 5hmC levels can be used as diagnostic criteria to distinguish between benign nevi and malignant melanomas [64]. Our study highlights the potential of cytosine modifications as biomarkers of cancerous cell proliferation, but questions whether colon cancer is suited to the recently described potential therapeutic avenue to restore TET activity [32].

## Materials and methods

### Patient samples

Research was conducted under the principles of the World Medical Association Helsinki agreement. Ethical approval was obtained from the Cambridgeshire Local Research Ethics Committee (LREC references 04/Q0108/125 and 06/Q0108/307). Forty-seven normal samples (composed of 16 samples taken more than 20 cm away from tumours (normal away (NA)) and 31 samples taken close to tumours (normal close (NC)), 36 adenoma (Ad) and 31 adenocarcinoma (T). Samples for tissue microarrays are described [65].

### Antibodies

Anti-5hmC rabbit polyclonal (Active Motif, 39791), anti-TET1 (SantaCruz sc-163443), anti-TET2 for western and IHC (Abcam ab94580), anti-TET2 for ChIP-seq (Santa Cruz sc-136926), anti-TET3 (Abnova), anti-Lamin B1 (Abcam ab16048), anti-beta Tubulin (Sigma T0198).

### hmeDIP-seq

Illumina libraries were prepared before the pull-down using 1 to 3 micrograms of sonicated genomic DNA (Bioruptor). Libraries were prepared using a ‘with-bead’ procedure [66] or with the TruSeq DNA sample preparation kit (Illumina) following manufacturer’s instructions. Adaptor modified genomic DNA was then immunoprecipitated following [67]. Input and pull-down material was whole genome amplified as previously described [68] except that samples were amplified with 10 PCR cycles, ran on 2% EX agarose gels (LifeTechnologies), size selection of 300 to 500 bp fragments with the MinElute gel extraction kit (Qiagen), further amplified with seven PCR cycles and purified with AMPURE XP beads. This procedure was also done for amplification with TruSeq reagents. Libraries were qualified and quantified by Bioanalyzer and submitted for sequencing by the CI Genomics core facility.

### Bioinformatic analysis - hmeDIP-seq

Illumina sequencing reads were aligned against the hg18 genome assembly using BWA. Mean read coverage around TSS was calculated using ‘GenomicRanges’ and ‘Rsamtools’ (Bioconductor). Read coverage was normalised per million mapped reads, subtracted from input and mean TSS coverage plotted. Feature Enrichment analysis was performed by using Rsamtools to count reads within feature locations obtained from Ensembl (hg18, May 2009). Promoter CpG content classification was as described in [67]. For gene ontology, functional enrichment of selected gene sets was assessed by fisher exact tests with and without graph correction using the ‘TopGO’ Bioconductor package [69]. False discovery rates were calculated by Benjamini Hochberg correction using R [70]. For genome wide repeats analysis, repeat scores were obtained by alignment of hmeDIP-seq reads to a repeat genome obtained by concatenating repeat locations annotated in Ensembl. ‘DiffBind’ [71] package was used to quantitatively compare 5hmC within peaks in normal and tumours. Heatmaps for comparison to meDIP-seq used ‘SeqMiner’ [72] and data kindly provided by C. Bock [43].

### Expression microarrays

Normal (n = 4), adenoma (n = 7) and adenocarcinoma (tumour, n = 14) mRNA were profiled using Illumina HumanWG6-V2 chips. The raw data were summarised using BeadStudio version 3.1.7, without background correction and imported into R using the ‘beadarray’ package [73] in Bioconductor. After quality control, arrays were background corrected using a normal-exponential model and then quantile normalised [74]. Illumina probes were annotated using the illuminaHumanv2.db Bioconductor package and poorly annotated probes were excluded prior to differential expression analysis [75]. A linear modelling approach was used to estimate the expression of each probe in normal, adenoma, and adenocarcinoma groups. Differential expression statistics were generated following empirical Bayes’ shrinkage of variances [76]. Illumina expression arrays validation used the 96.96 Biomark Dynamic Array platform (Fluidigm) and Taqman assays (ABI) following manufacturer’s instructions (Fluidigm). Fold change between normal and tumour was calculated by the delta Ct method using *B2M*, *HPRT1*, *SDHA* or *PSMC4* as normaliser loci (Additional file 14 and list of assays in Additional file 1). Expression values for loci identified as TET2 targets by ChIP-seq in HCT116 cells were obtained from GSE36133.

### Mass spectrometry

1 μg of genomic DNA was incubated with 5 U of DNA Degradase Plus (Zymo Research) at 37°C for 3 h and filtered through Amicon 10 kDa centrifugal filter units (Millipore). The concentrations of 2′-deoxycytidine, 5-methyl-2′-deoxycytidine and 5-hydroxymethyl-2′-deoxycytidine in the filtrate were determined using an AB Sciex Triple Quad 6500 mass spectrometer fitted with an Agilent Infinity 1290 LC system and an Acquity UPLC HSS T3 column. The global levels of mC and hmC were expressed as percentages over total 2′-deoxycytidines.

### CRC TMA IF

After epitope retrieval by boiling in an EDTA solution the slide was rinsed in PBS and blocked. Primary (anti-5hmC and anti-5mC) and secondary (Alexa647 anti-rabbit and Alexa448 anti-mouse (Invitrogen)) antibodies were sequentially applied for 1 h each with 3× washes of PBS/0.1% Tween 20 in between. After a final 3× washes the slide was mounted with DAPI and scanned onto the Ariol system for analysis.

### qRT-PCR

For Figure 2, 1 μg total RNA was treated with 1U DNaseI (Promega 9PMIM610) and cDNA prepared with SuperscriptIII reverse transcriptase (Invitrogen) and random primers. Targets were quantified with 1× Fast Sybr (ABI) and 1× Quantitect assays (Qiagen) by the standard curve method using serial dilutions of cDNA template from Jeg3 cells and normalised to *B2M*. For supplementary Figure 3, 1 μg RNA was reverse transcribed using Quantitect reverse transcription kit (Qiagen) following manufacturer’s instructions. Real-time PCR used the 96.96 Biomark Dynamic Array platform (Fluidigm) and Taqman assays (ABI) following manufacturer’s instructions (Fluidigm). Fold change between normal and tumour was calculated by the delta Ct method using *B2M* as the normaliser. All expression assays are listed in Additional file 1.

### Infinium27k and 450 k

Bisulfite-converted DNA (EZ DNA Methylation-Gold, Zymo Research) was analysed using Illumina Infinium HumanMethylation27 BeadChips in the Cambridge Genomic Services, Cambridge University, UK. Data were analysed using BeadStudio (Illumina, Inc.) and R. The locus methylation was calculated as the log ratio of the Unmethylated and Methylated channels, and a standard error of the log-ratio was estimated. Models were fitted with limma [77] using weights derived from the standard errors. Separate analysis were performed for loci measured in the red channel and loci measured in the green channel. Infinium450k for HCT116 cells in Figure 4 used beta values from GSE29290 for Infinium ID annotations obtained using the ‘IlluminaHumanMethylation450k.db’ package (Bioconductor) in R. For the 450 k analysis of TET2 shRNA knockdowns, raw Infinium data were filtered by removing low quality data using a detection *P* value threshold of 0.05. Cross-reactive probes (that is, targeting several genomic locations) and probes containing SNPs were filtered out using the extended annotation provided by Price *et al.* [78] (see [79] for a detailed description). Probes associated to X and Y chromosomes were removed from the analysis. Beta-values were computed using the formula Beta-value = M/[U + M] where M and U are the raw ‘methylated’ and ‘unmethylated’ signals, respectively. Beta values were corrected for type I and type II bias using the peak-based correction [79,80].

### ChIP-seq

ChIP-seq for TET2 was performed as previously described [63].

### shRNA

Stable knockdown of TET2 in HCT116 used MISSION shRNA Lentiviral Transduction Particles (SHCLNV-NM_017628, Sigma) following manufacturer’s instructions and selection with 2 ug/mL Puromycin (A11138-03, Life Technologies). For TET2C 10MOI of TRCN0000418976 and for TET2 + 3 5MOI each of TRCN0000418976 and TRCN0000246258 were used. shRNA control used 10MOI of MISSION pLKO.1-puro Non-Target shRNA Control Transduction Particles (SHC016V-1EA, Sigma).

### Additional methods

Additional methods can be found in Additional file 15.

## Additional files

Additional file 1:
**Summarising 5hmC promoter types, loci that gain and lose methylation in colon cancer, HCT116 TET2 target promoters, qRT-PCR assays, Gluc-MS-qPCR primers, hmeDIP-qPCR primers, TET2 ChIP-seq qPCR validation primers, TETs targeted sequencing primers, our colon cancer cohort and sequencing reads statistics.**
Additional file 2: Figure S1. Gene ontology analysis for 5-hmC enriched narrow and broad promoters in normal human colon. 5-hmC profiles for the top 5 loci of each promoter type. **(a)** Top 20 biological processes ranked by *P* value that associated with 5-hmC ‘narrow’ promoters (top 1,000 ranked by 5-hmC content). The GO term name is followed by the GO identifier and the term depth. Plotted alongside the *P* value for the term in ‘narrow’ promoters is the value for that term in ‘broad’ promoters; this to identify terms that are unique or shared. The dashed vertical line represents the -log10 of *P* <0.05. **(b)** As in (a) but for ‘broad’ promoters. ‘Narrow’ promoters were enriched for membrane transport processes whereas ‘broad’ promoters were enriched for cell differentiation, developmental processes and morphogenesis. **(c)** 5-hmC profiles for the top five ‘narrow’ promoters. Each frame is 6 kb (-3 kb to +3 kb of the TSS). Orange bars are the CpG islands (UCSC). 5-hmC is highly enriched in the upstream shore of *ACTN2*, *CALY* and *ARSA* promoter CpG islands, whereas 5-hmC spans across the promoter CpG island and TSS of *RTKN. RPS8* showed enrichment for 5-hmC in the gene body where the promoter for the SNORD38 gene is present (not displayed). High content of 5-hmC is also present in the downstream shore of CALY and ARSA promoter CpG islands. **(d)** 5-hmC profiles for the top five ‘broad’ promoters where the content of 5-hmC is similar between the upstream and downstream shores. Additional file 3: Figure S2. Comparison of hmeDIP-seq to meDIP-seq along with 5-hmC promoter profiles, promoter CpG content and Infinium methylation estimates in normal colon. **(a)** SeqMiner [72] heatmaps of hmeDIP-seq (this study) and meDIP-seq (from [43]) clustered by enrichment profiles from -3 kb to +20 kb around the TSS. Ten clusters were generated. Except for cluster 2, where a 5-mC-specific enrichment is observed at the TSS, the enrichment profiles around genes are similar between 5-hmC and 5-mC. **(b)** Further comparison of 5-hmC and 5-mC profiles across all loci from -3 kb to +3 kb around the TSS illustrates differences in enrichment patterns at the TSS and upstream promoter region. **(c)** 5-hmC ‘narrow’ promoters (see main Figure 1) are typified in clusters 3 and 8 whereas ‘broad’ promoters belong to clusters 5, 6, 7 and 9. **(d)** Proportions of promoters classified by their CpG content (high (HCP), intermediate (ICP), low (LCP)) in the 10 clusters. Cluster 2 is highly enriched for LCP. **(e)** Comparison of DNA methylation levels by meDIP-seq from [9] to the Infinium arrays from this study. **(f)** The distance of the Infinium probes to the TSS for loci plotted in the heatmaps in (a). Collectively, these results show that 5-hmC profiles are distinct to 5-mC profiles at a subset of promoter regions and that the Inifinium arrays recapitulate meDIP-seq enrichment patterns at the TSS in normal colon. Additional file 4: Figure S3. TETs levels do not correlate with the global change of 5-hmC in colon tumours. **(a)** Expression levels of selected loci in 15 normal-tumour matched samples by Taqman qRT-PCR. Dashed horizontal lines are at 0.6 log2 (approximately 1.5-fold linear). **(b)** Correlation of changes in global 5-hmC levels (by LCMS) to the changes in TETs expression levels in the 15 matched cases. Additional file 5: Figure S4. TETs mutation screen. **(a)** Plot of sequence coverage over amino acid residue triplets in the *α*-KG and Fe2 binding sites where mutation was not detected in 36 normals, 38 adenomas, 28 adenocarcinomas or HCT116 cells. Vertical dashed line represents a coverage of 10×. **(b)** Global levels of 5-hmC did not correlate with non-synonymous mutations identified in *TET*s DSBH (catalytic) domain in colon normal and cancer samples. ‘Wild-type’ (WT), heterozygous (Het), homozygous (Hom) and not determined (ND) are indicated. Most amino acid changes have been identified as common variants (1,000 genomes and ESP) except TET3_R1609H and TET2_G1936D. Primer sequences are listed in Additional file 1. Additional file 6: Figure S5. Content of 5-hmC at repetitive elements in normal human colon and in tumours. **(a)** Plotted are the percentages of hmeDIP-seq reads in each repeat class in the bound (Bnd) and input (Inp) fractions in normal and tumour DNA. All repeat types are enriched for 5-hmC over input in normal tissues. Note that the ratio of Bnd to Inp in tumours is only slightly reduced in Simple, Tandem, Dust and LTR repeats. This ratio is clearly reduced in LINE-1, Low Complexity, SINE, Type II transposons and the less abundant Other, Unknown and satellite repeats. The mean and standard error of the mean are indicated. **(b, c)** hmeDIP-qPCR analysis of the 5’ end of LINE-1 elements and of alpha satellite DNA. NA is normal tissue away from the tumour, NC is normal tissue close to the tumours, Ad is adenoma and T is adenocarcinoma. 5-hmC was enriched at the 5’ end of LINE-1 elements in NA and NC and reduced in Ad and T. Alpha satellite showed no enrichment of 5-hmC in any tissue but showed high levels of DNA methylation in normal tissues and methylation loss in tumours. Additional file 7: Figure S6. Distribution of 5-hmC in adenocarcinomas and the correlation of reduced 5-hmC with gene activity in neoplastic tissue. **(a)** Quantification of 5-hmC enrichments across genomic features. **(b)** Comparison of 5-hmC distribution patterns around the TSS (-3 kb to +20 kb of TSS) in normals and tumours. **(c)** Validation of the changes in 5-hmC content in normal and tumour DNA at selected promoter and gene body loci by Gluc-MS-qPCR. 5-hmC-specific glycosylation by the T4 glucosyltransferase inhibits MspI endonuclease activity. The plot shows the fold change of MspI restriction activity in T4 glucosyltransferase-treated DNA relative to non-glycosylated DNA normalised to a locus lacking an MspI site as a loading control (vertical dashed line). 5-hmC is clearly reduced but not absent in tumours. Primers used are listed in Additional file 1. **(d)** Expression levels of genes with ‘narrow’ and ‘broad’ promoters in normals (N) and tumours (T). The marked drop in 5-hmC does not grossly hinder the transcriptome. However a small, but significant, decrease in activity was observed for genes with ‘broad’ 5-hmC promoters (*P* values were calculated by a Wilcox test). Additional file 8: Figure S7. The reciprocal pattern of 5-hmC high/low in normal with 5-mC loss/gain in tumours is already present in adenomas and is observed in islands, island shores and colon cancer specific DMRs. Infinium450k annotation was used to identify CpGi relation of the Infinium27k probes. **(a)** In adenomas and **(b)** adenocarcinomas, loci with high content of 5-hmC in normal lose methylation in neoplastic tissues whereas those with a low content of 5-hmC more likely gain methylation. *P* values were calculated by a limma geneSetTest. **(c)** The reciprocal behaviour of 5-hmC high/low with 5-mC loss/gain is observed at islands, island shores or distant sites (shelves). Notably, 5-mC loss was more frequent at shores but more pronounced at distant sites. On the other hand, 5-mC gain was more frequent and more pronounced at islands. The numbers of probes for each box are indicated above the plot. **(d)** The small differentially methylated regions (sDMR) that gain methylation in cancer (data from [8]) are less enriched for 5-hmC in normal tissue relative to All sDMRs whereas those that lose 5-mC in cancer have higher levels of 5-hmC in normal tissue. sDMRs cover a relatively small portion of the genome. **(e)** The large blocks that lose methylation in cancer and span over 60% of the genome [8] showed depletion of 5-hmC relative to the input in our normal tissue hmeDIP-seq. Blocks that were shown to gain methylation in cancer and span approximately 1% of the genome di however show an enrichment of 5-hmC reads over input in our normal tissues. Additional file 9: Figure S8. Comparison of 5-hmC in normal human colon to 5-mC in normal and tumour at selected WNT pathway promoters. **(a)** Heatmap for a selected panel of WNT pathway components. The level of 5-hmC in normal (undetected at the 0 vertical dashed line) is compared to the level of 5-mC in normal (0.5 vertical dashed line = 50% methylation) and to the fold change in methylation in tumours (gain of methylation is to the right of the 0 dashed line). Gene symbols and Infinium27k IDs are shown on the right together with the *P* values for the change in methylation in tumours. **(b)** Validation of the Infinium arrays by bisulphite pyrosequencing for selected loci. The progressive gain of methylation (not shown in the heatmap in a) from normal to adenoma to adenocarcinoma can be observed. The low level of methylation in normal by bisulphite pyrosequencing confirms the low level of 5-hmC in normal by hmeDIP-seq. Additional file 10: Figure S9. Persistent presence of 5-hmC at promoters in tumours and increased propensity to DNA methylation loss. Plot comparing the mean methylation in normals to the methylation change in tumours (n = 17 matched pairs). Each black dot is a single CpG and those highlighted in grey showed a *P* value <0.01 for the methylation change. Red points indicate presence of hmeDIP-seq reads above 4 in a 200 bp window around the Infinium probe. DNA methylation was lost in 27% of gene promoters that retain 5-hmC in tumour, whereas only 8.5% gained methylation. Additional file 11: Figure S10. HCT116 cells have very low global levels of 5-hmC with maintained expression of TETs. **(a, b)** LCMS measurement of the global content of 5-hmC and 5-mC in our colon cancer cohort, in HCT116 cells and mES cells genomic DNA. **(c)** Comparison of *TET*s transcripts levels between primary tissues (N = normal, Ad = adenoma, T = adenocarcinoma) and those in HCT116 cells. Primer sequences are listed in Additional file 1. **(d)** Western blot for TETs and LaminB1 in HCT116 cells total cell extract (T), cytoplasmic fraction (C) and nuclear fraction (N). **(e)** Immunohistochemistry for TET2 in HCT116 cell pellet. Scale bar is at 20 μm. Additional file 12: Figure S11. TET2 protein is present in normal and adenocarcinoma tissues. Immunohistochemistry for TET2 in normal colon **(a to d)** and colon adenocarcinoma **(e, f)**. TET2 is present mainly in the cytoplasm of cryptal epithelium with occasional cells showing both diffuse strong nuclear and cytoplasmic staining pattern **(c, d)**. Predominant cytoplasmic staining is observed in tumours **(g, h)**. Scale bars are at 200 μm (for **a**, **b**, **e**, **f**) or 20 μm (for **c**, **d**, **g**, **h**). Additional file 13: Figure S12. qPCR Validation of ChIP-seq for TET2 in HCT116 cells. ChIPs using antibodies to TET2 (sc-136926; Santa Cruz) or IgG (sc-136926; Santa Cruz) were performed on the indicated targets (primer sequences are available in Additional file 1). ‘% Input’ represents real-time qPCR values normalised with respect to the input chromatin. Values are represented as means of two independent biological experiments. Asterisks indicate 11 out of 15 loci analysed showed TET2-specific enrichment. Additional file 14: Figure S13. Validation of the Illumina expression arrays by Taqman qRT-PCR. **(a)** Plot comparing the fold change in expression from normal to tumour reported by the Illumina arrays and the fold change reported by Taqman qRT-PCR normalised to *B2M*. Each value from the Illumina arrays used four normals and 14 tumours. Each Taqman value is the average of quadruplicate measurements from 13 normal-tumour pairs. **(b** to **d)** As in (a) but normalised to *HPRT1*, *SDHA* or *PSMC4*, respectively. **(e)** Boxplot of the fold change for the individual assays ordered by their mean. Each assay contains 52 values from quadruplicate measurements of 13 normal-tumour pairs. Taqman assays are listed in Additional file 1. Additional file 15:
**Supplementary information.**
