# Supplementary figures and images for: 5-hydroxymethylcytosine marks promoters in colon that resist DNA hypermethylation in cancer
Source: Genome Biol. 2015 Apr 1;16(1):69. doi: 10.1186/s13059-015-0605-5 (PMC4380107; doi:10.1186/s13059-015-0605-5)

Uribe-Lewis\_Additional file 2\_Supplementary Fig. 1.

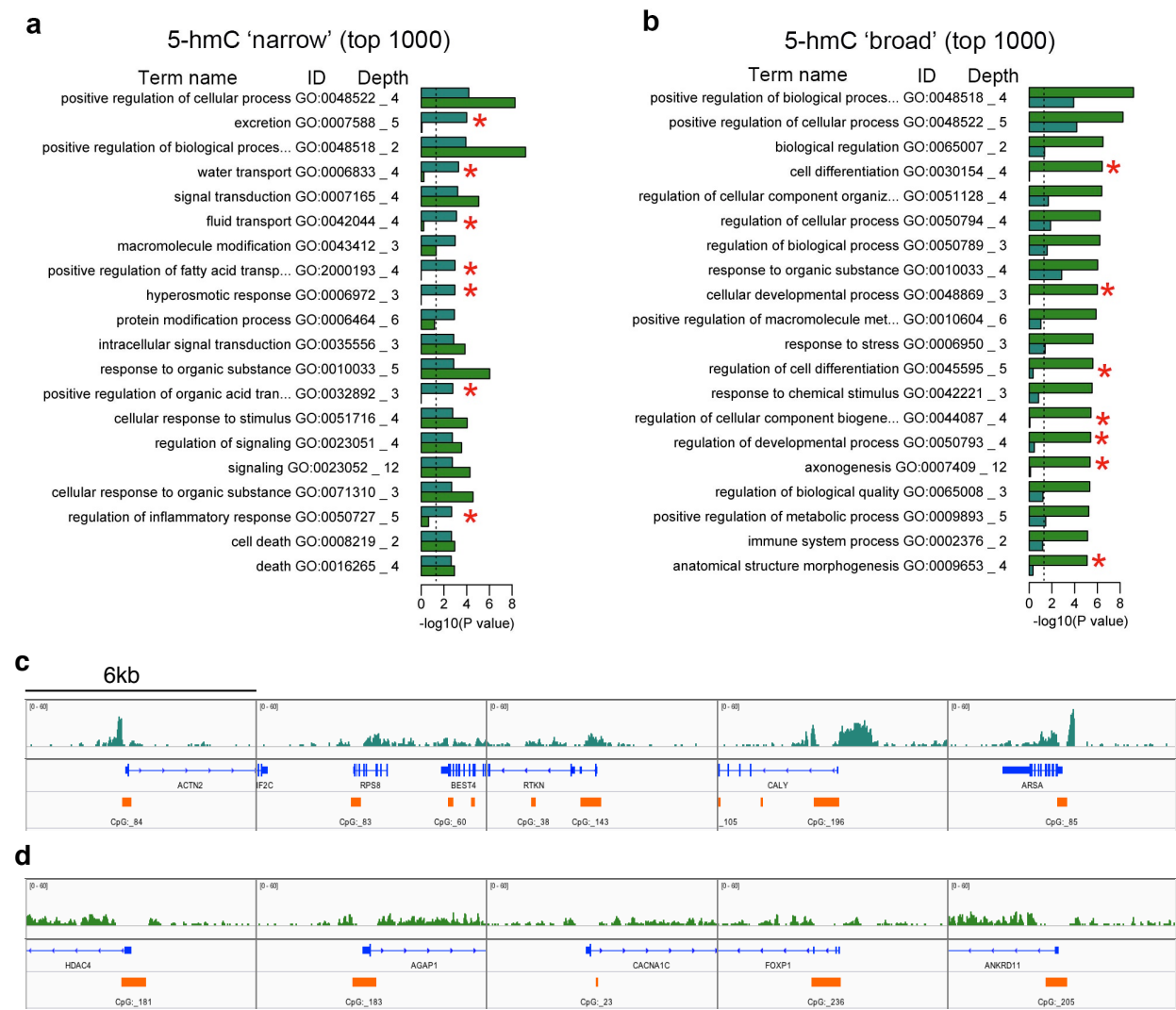

Supplement: Additional file 2: Figure S1. — Gene ontology analysis for 5-hmC enriched narrow and broad promoters in normal human colon. 5-hmC profiles for the top 5 loci of each promoter type. (a) Top 20 biological processes ranked by P value that associated with 5-hmC ‘narrow’ promoters (top 1,000 ranked by 5-hmC content). The GO term name is followed by the GO identifier and the term depth. Plotted alongside the P value for the term in ‘narrow’ promoters is the value for that term in ‘broad’ promoters; this to identify terms that are unique or shared. The dashed vertical line represents the -log10 of P <0.05. (b) As in (a) but for ‘broad’ promoters. ‘Narrow’ promoters were enriched for membrane transport processes whereas ‘broad’ promoters were enriched for cell differentiation, developmental processes and morphogenesis. (c) 5-hmC profiles for the top five ‘narrow’ promoters. Each frame is 6 kb (-3 kb to +3 kb of the TSS). Orange bars are the CpG islands (UCSC). 5-hmC is highly enriched in the upstream shore of ACTN2, CALY and ARSA promoter CpG islands, whereas 5-hmC spans across the promoter CpG island and TSS of RTKN. RPS8 showed enrichment for 5-hmC in the gene body where the promoter for the SNORD38 gene is present (not displayed). High content of 5-hmC is also present in the downstream shore of CALY and ARSA promoter CpG islands. (d) 5-hmC profiles for the top five ‘broad’ promoters where the content of 5-hmC is similar between the upstream and downstream shores. [file 13059_2015_605_MOESM2_ESM.pdf]

Uribe-Lewis\_Additional file 3\_Supplementary Fig. 2.

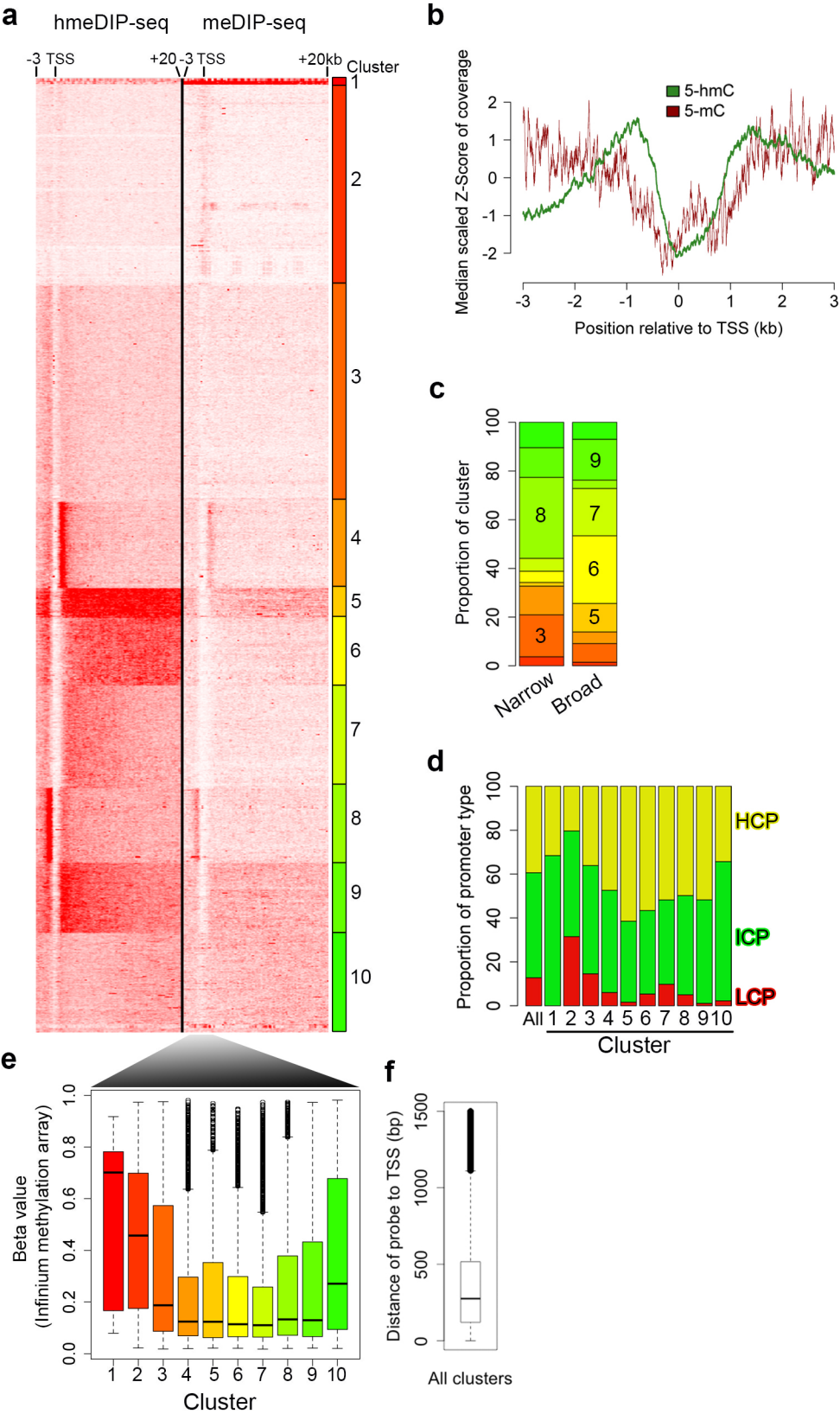

Supplement: Additional file 3: Figure S2. — Comparison of hmeDIP-seq to meDIP-seq along with 5-hmC promoter profiles, promoter CpG content and Infinium methylation estimates in normal colon. (a) SeqMiner [72] heatmaps of hmeDIP-seq (this study) and meDIP-seq (from [43]) clustered by enrichment profiles from -3 kb to +20 kb around the TSS. Ten clusters were generated. Except for cluster 2, where a 5-mC-specific enrichment is observed at the TSS, the enrichment profiles around genes are similar between 5-hmC and 5-mC. (b) Further comparison of 5-hmC and 5-mC profiles across all loci from -3 kb to +3 kb around the TSS illustrates differences in enrichment patterns at the TSS and upstream promoter region. (c) 5-hmC ‘narrow’ promoters (see main Figure 1) are typified in clusters 3 and 8 whereas ‘broad’ promoters belong to clusters 5, 6, 7 and 9. (d) Proportions of promoters classified by their CpG content (high (HCP), intermediate (ICP), low (LCP)) in the 10 clusters. Cluster 2 is highly enriched for LCP. (e) Comparison of DNA methylation levels by meDIP-seq from [9] to the Infinium arrays from this study. (f) The distance of the Infinium probes to the TSS for loci plotted in the heatmaps in (a). Collectively, these results show that 5-hmC profiles are distinct to 5-mC profiles at a subset of promoter regions and that the Inifinium arrays recapitulate meDIP-seq enrichment patterns at the TSS in normal colon. [file 13059_2015_605_MOESM3_ESM.pdf]

**a**

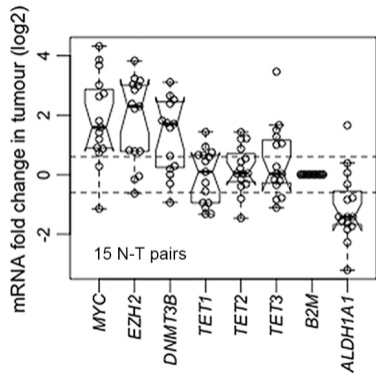

**b**

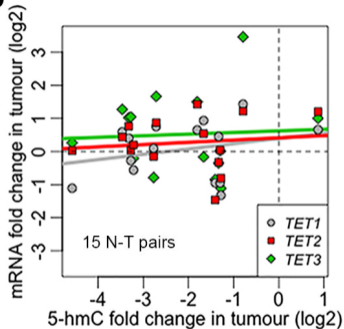

Supplement: Additional file 4: Figure S3. — TETs levels do not correlate with the global change of 5-hmC in colon tumours. (a) Expression levels of selected loci in 15 normal-tumour matched samples by Taqman qRT-PCR. Dashed horizontal lines are at 0.6 log2 (approximately 1.5-fold linear). (b) Correlation of changes in global 5-hmC levels (by LCMS) to the changes in TETs expression levels in the 15 matched cases. [file 13059_2015_605_MOESM4_ESM.pdf]

Uribe-Lewis\_Additional file 5\_Supplementary Fig. 4.

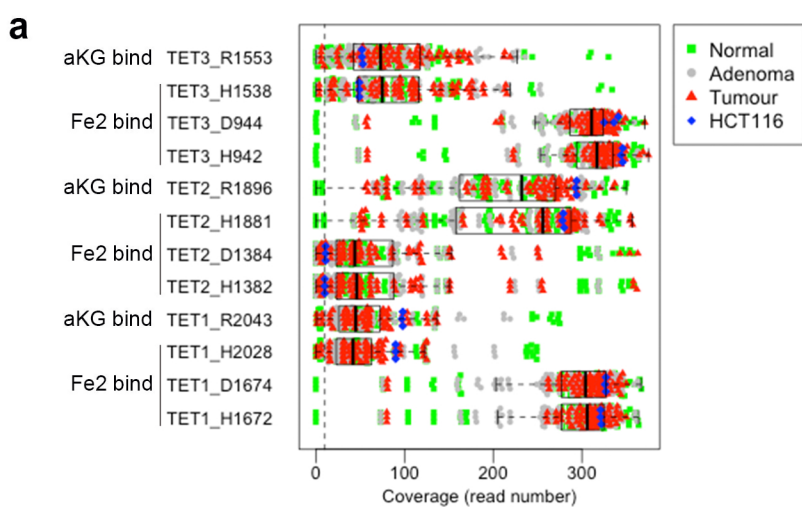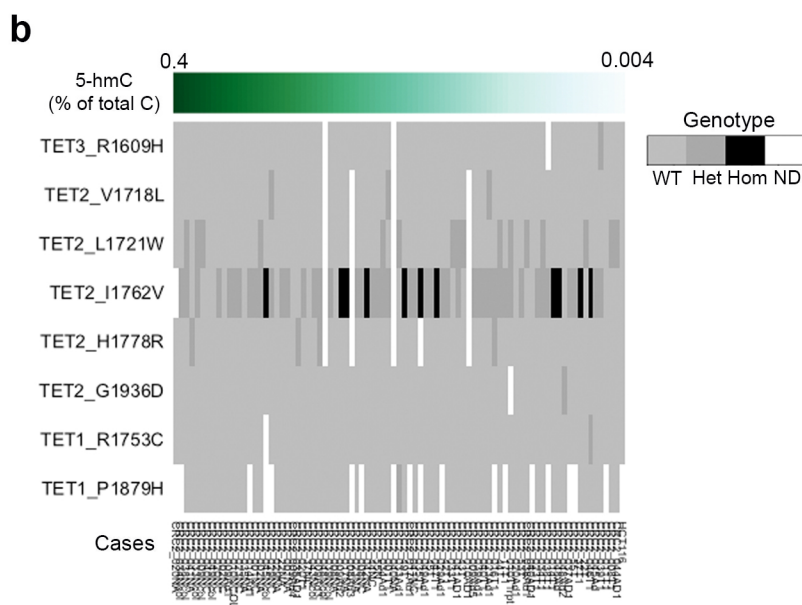

Supplement: Additional file 5: Figure S4. — TETs mutation screen. (a) Plot of sequence coverage over amino acid residue triplets in the α-KG and Fe2 binding sites where mutation was not detected in 36 normals, 38 adenomas, 28 adenocarcinomas or HCT116 cells. Vertical dashed line represents a coverage of 10×. (b) Global levels of 5-hmC did not correlate with non-synonymous mutations identified in TETs DSBH (catalytic) domain in colon normal and cancer samples. ‘Wild-type’ (WT), heterozygous (Het), homozygous (Hom) and not determined (ND) are indicated. Most amino acid changes have been identified as common variants (1,000 genomes and ESP) except TET3_R1609H and TET2_G1936D. Primer sequences are listed in Additional file 1. [file 13059_2015_605_MOESM5_ESM.pdf]

Uribe-Lewis\_Additional file 6\_Supplementary Fig. 5.

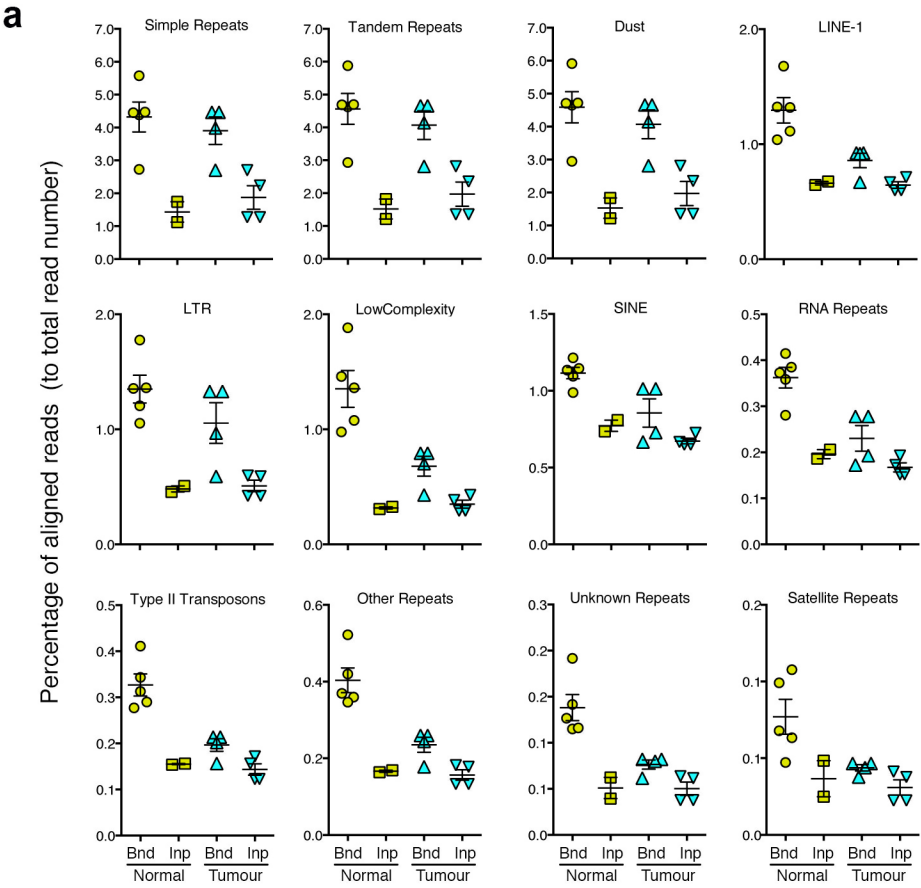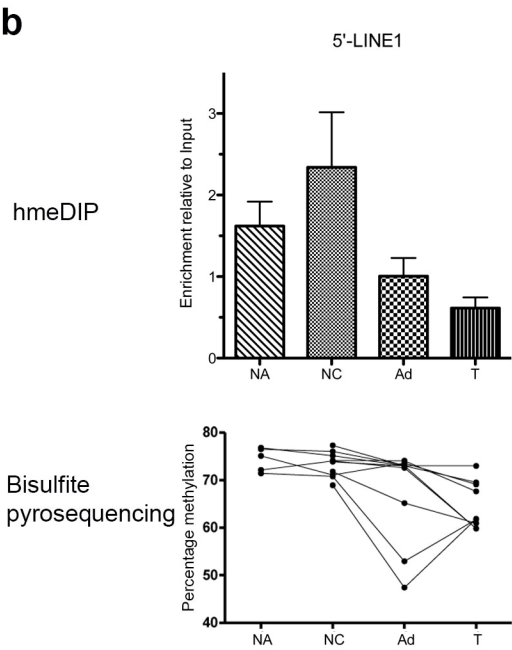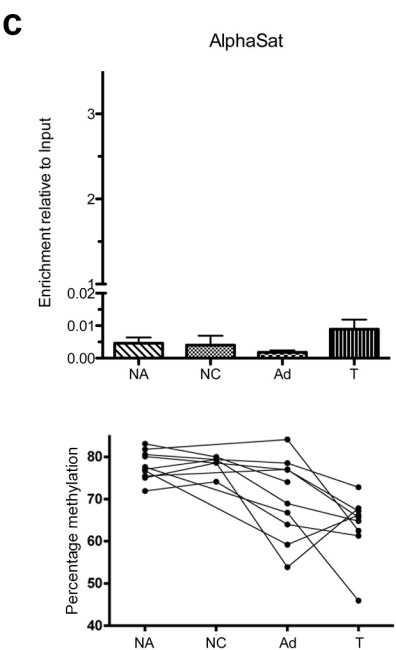

Supplement: Additional file 6: Figure S5. — Content of 5-hmC at repetitive elements in normal human colon and in tumours. (a) Plotted are the percentages of hmeDIP-seq reads in each repeat class in the bound (Bnd) and input (Inp) fractions in normal and tumour DNA. All repeat types are enriched for 5-hmC over input in normal tissues. Note that the ratio of Bnd to Inp in tumours is only slightly reduced in Simple, Tandem, Dust and LTR repeats. This ratio is clearly reduced in LINE-1, Low Complexity, SINE, Type II transposons and the less abundant Other, Unknown and satellite repeats. The mean and standard error of the mean are indicated. (b, c) hmeDIP-qPCR analysis of the 5’ end of LINE-1 elements and of alpha satellite DNA. NA is normal tissue away from the tumour, NC is normal tissue close to the tumours, Ad is adenoma and T is adenocarcinoma. 5-hmC was enriched at the 5’ end of LINE-1 elements in NA and NC and reduced in Ad and T. Alpha satellite showed no enrichment of 5-hmC in any tissue but showed high levels of DNA methylation in normal tissues and methylation loss in tumours. [file 13059_2015_605_MOESM6_ESM.pdf]

Uribe-Lewis\_Additional file 7\_Supplementary Fig. 6.

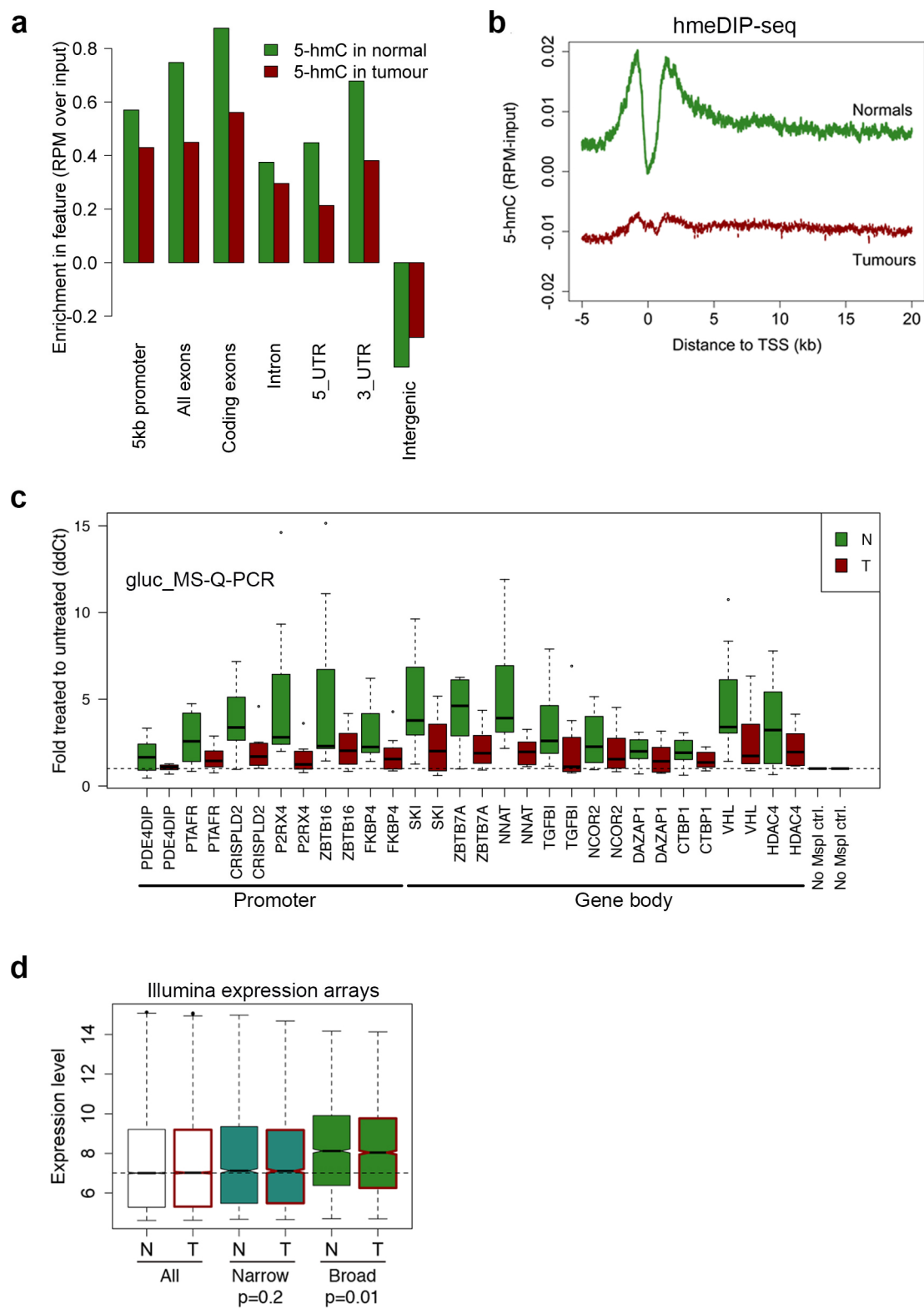

Supplement: Additional file 7: Figure S6. — Distribution of 5-hmC in adenocarcinomas and the correlation of reduced 5-hmC with gene activity in neoplastic tissue. (a) Quantification of 5-hmC enrichments across genomic features. (b) Comparison of 5-hmC distribution patterns around the TSS (-3 kb to +20 kb of TSS) in normals and tumours. (c) Validation of the changes in 5-hmC content in normal and tumour DNA at selected promoter and gene body loci by Gluc-MS-qPCR. 5-hmC-specific glycosylation by the T4 glucosyltransferase inhibits MspI endonuclease activity. The plot shows the fold change of MspI restriction activity in T4 glucosyltransferase-treated DNA relative to non-glycosylated DNA normalised to a locus lacking an MspI site as a loading control (vertical dashed line). 5-hmC is clearly reduced but not absent in tumours. Primers used are listed in Additional file 1. (d) Expression levels of genes with ‘narrow’ and ‘broad’ promoters in normals (N) and tumours (T). The marked drop in 5-hmC does not grossly hinder the transcriptome. However a small, but significant, decrease in activity was observed for genes with ‘broad’ 5-hmC promoters (P values were calculated by a Wilcox test). [file 13059_2015_605_MOESM7_ESM.pdf]

Uribe-Lewis\_Additional file 8\_Supplementary Fig. 7.

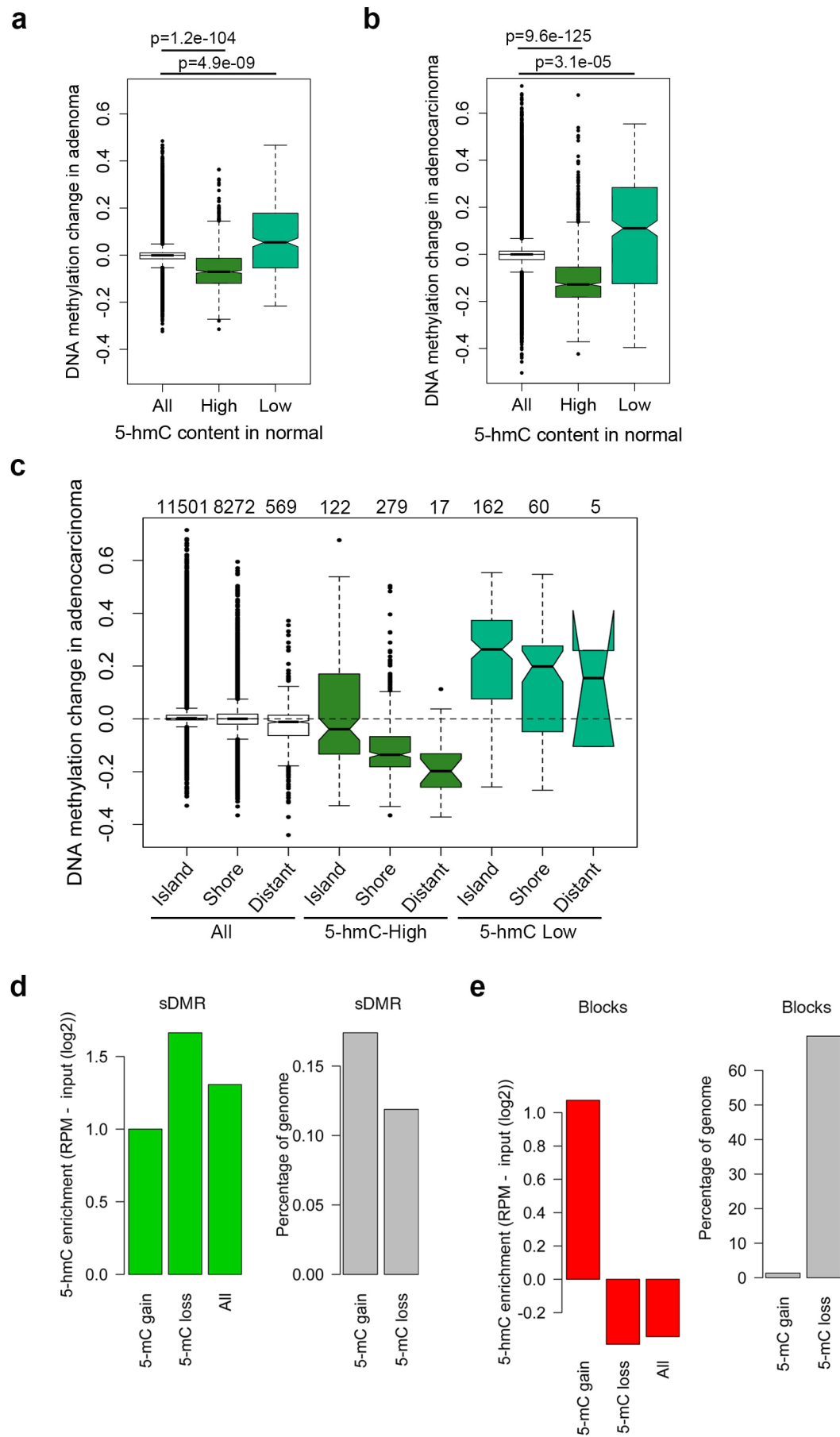

Supplement: Additional file 8: Figure S7. — The reciprocal pattern of 5-hmC high/low in normal with 5-mC loss/gain in tumours is already present in adenomas and is observed in islands, island shores and colon cancer specific DMRs. Infinium450k annotation was used to identify CpGi relation of the Infinium27k probes. (a) In adenomas and (b) adenocarcinomas, loci with high content of 5-hmC in normal lose methylation in neoplastic tissues whereas those with a low content of 5-hmC more likely gain methylation. P values were calculated by a limma geneSetTest. (c) The reciprocal behaviour of 5-hmC high/low with 5-mC loss/gain is observed at islands, island shores or distant sites (shelves). Notably, 5-mC loss was more frequent at shores but more pronounced at distant sites. On the other hand, 5-mC gain was more frequent and more pronounced at islands. The numbers of probes for each box are indicated above the plot. (d) The small differentially methylated regions (sDMR) that gain methylation in cancer (data from [8]) are less enriched for 5-hmC in normal tissue relative to All sDMRs whereas those that lose 5-mC in cancer have higher levels of 5-hmC in normal tissue. sDMRs cover a relatively small portion of the genome. (e) The large blocks that lose methylation in cancer and span over 60% of the genome [8] showed depletion of 5-hmC relative to the input in our normal tissue hmeDIP-seq. Blocks that were shown to gain methylation in cancer and span approximately 1% of the genome di however show an enrichment of 5-hmC reads over input in our normal tissues. [file 13059_2015_605_MOESM8_ESM.pdf]

a

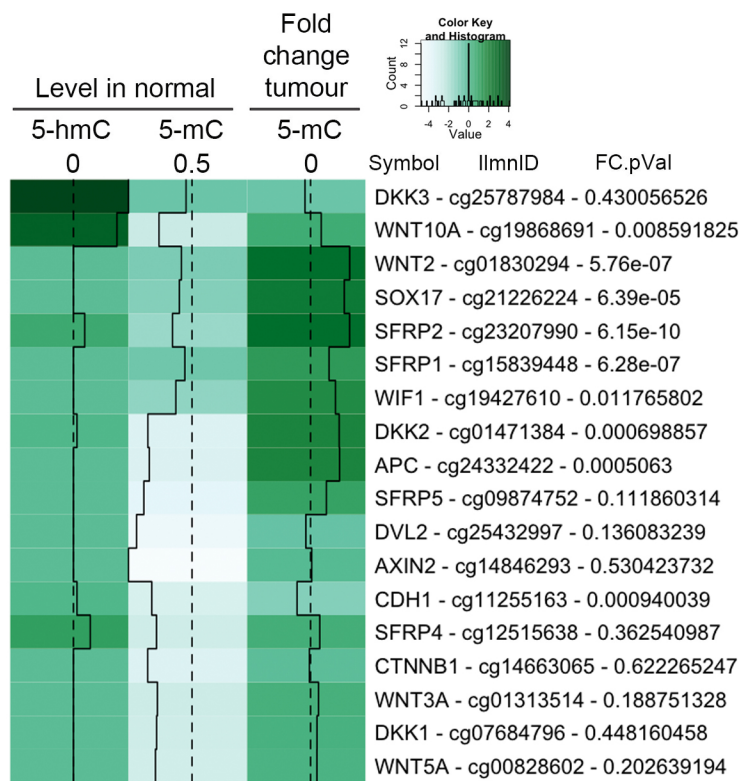

b

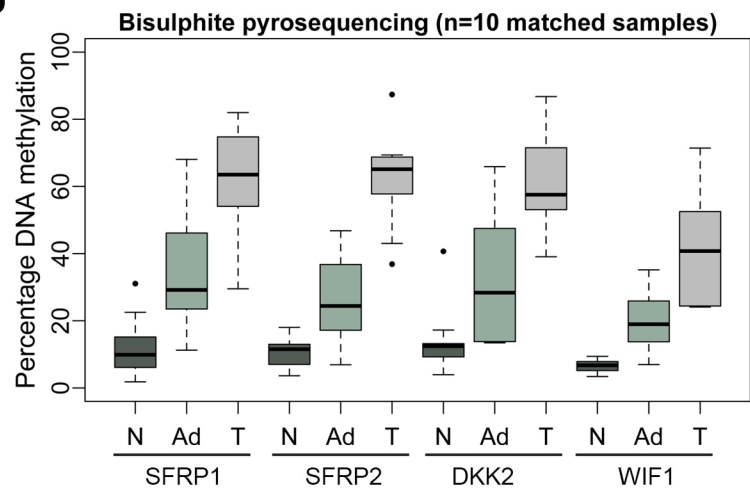

Supplement: Additional file 9: Figure S8. — Comparison of 5-hmC in normal human colon to 5-mC in normal and tumour at selected WNT pathway promoters. (a) Heatmap for a selected panel of WNT pathway components. The level of 5-hmC in normal (undetected at the 0 vertical dashed line) is compared to the level of 5-mC in normal (0.5 vertical dashed line = 50% methylation) and to the fold change in methylation in tumours (gain of methylation is to the right of the 0 dashed line). Gene symbols and Infinium27k IDs are shown on the right together with the P values for the change in methylation in tumours. (b) Validation of the Infinium arrays by bisulphite pyrosequencing for selected loci. The progressive gain of methylation (not shown in the heatmap in a) from normal to adenoma to adenocarcinoma can be observed. The low level of methylation in normal by bisulphite pyrosequencing confirms the low level of 5-hmC in normal by hmeDIP-seq. [file 13059_2015_605_MOESM9_ESM.pdf]

Uribe-Lewis\_Additional file 10\_Supplementary Fig. 9.

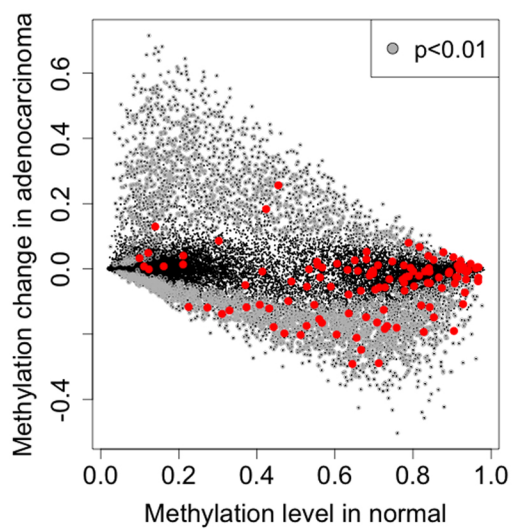

Supplement: Additional file 10: Figure S9. — Persistent presence of 5-hmC at promoters in tumours and increased propensity to DNA methylation loss. Plot comparing the mean methylation in normals to the methylation change in tumours (n = 17 matched pairs). Each black dot is a single CpG and those highlighted in grey showed a P value <0.01 for the methylation change. Red points indicate presence of hmeDIP-seq reads above 4 in a 200 bp window around the Infinium probe. DNA methylation was lost in 27% of gene promoters that retain 5-hmC in tumour, whereas only 8.5% gained methylation. [file 13059_2015_605_MOESM10_ESM.pdf]

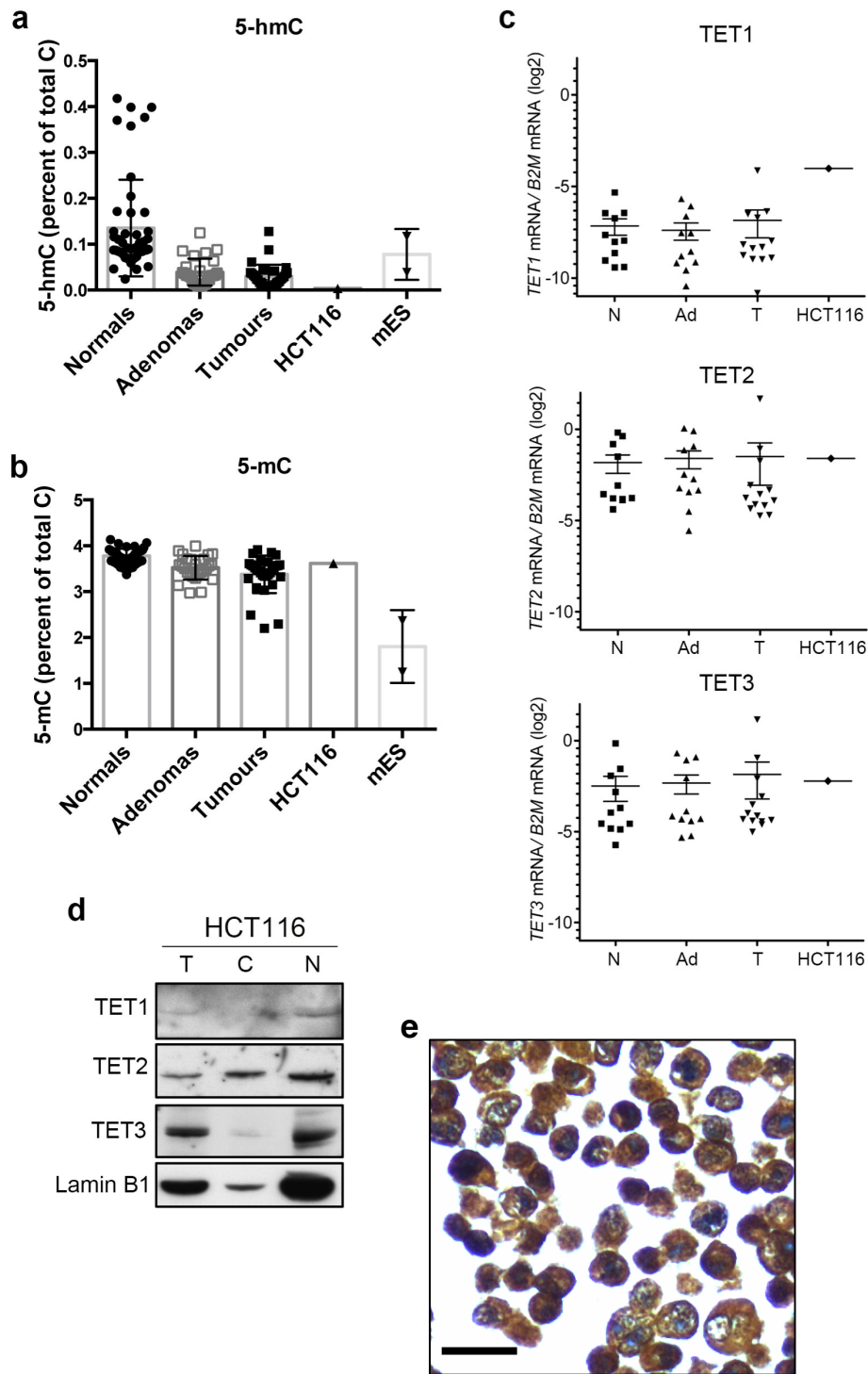

Supplement: Additional file 11: Figure S10. — HCT116 cells have very low global levels of 5-hmC with maintained expression of TETs. (a, b) LCMS measurement of the global content of 5-hmC and 5-mC in our colon cancer cohort, in HCT116 cells and mES cells genomic DNA. (c) Comparison of TETs transcripts levels between primary tissues (N = normal, Ad = adenoma, T = adenocarcinoma) and those in HCT116 cells. Primer sequences are listed in Additional file 1. (d) Western blot for TETs and LaminB1 in HCT116 cells total cell extract (T), cytoplasmic fraction (C) and nuclear fraction (N). (e) Immunohistochemistry for TET2 in HCT116 cell pellet. Scale bar is at 20 μm. [file 13059_2015_605_MOESM11_ESM.pdf]

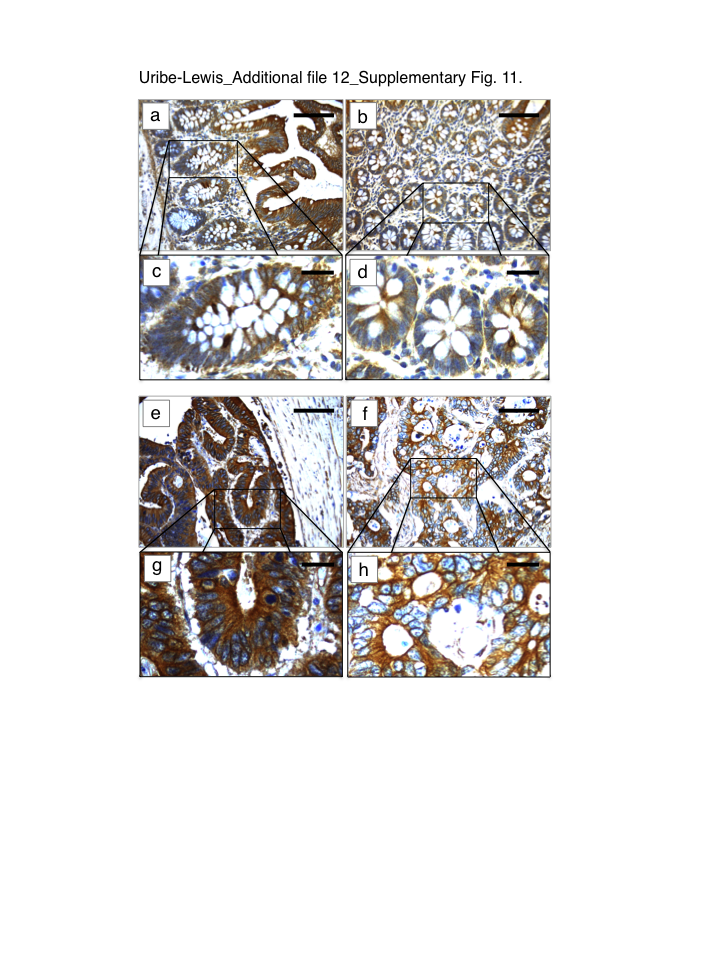

Supplement: Additional file 12: Figure S11. — TET2 protein is present in normal and adenocarcinoma tissues. Immunohistochemistry for TET2 in normal colon (a to d) and colon adenocarcinoma (e, f). TET2 is present mainly in the cytoplasm of cryptal epithelium with occasional cells showing both diffuse strong nuclear and cytoplasmic staining pattern (c, d). Predominant cytoplasmic staining is observed in tumours (g, h). Scale bars are at 200 μm (for a, b, e, f) or 20 μm (for c, d, g, h). [file 13059_2015_605_MOESM12_ESM.tif]

Uribe-Lewis\_Additional file 12\_Supplementary Fig. 11.

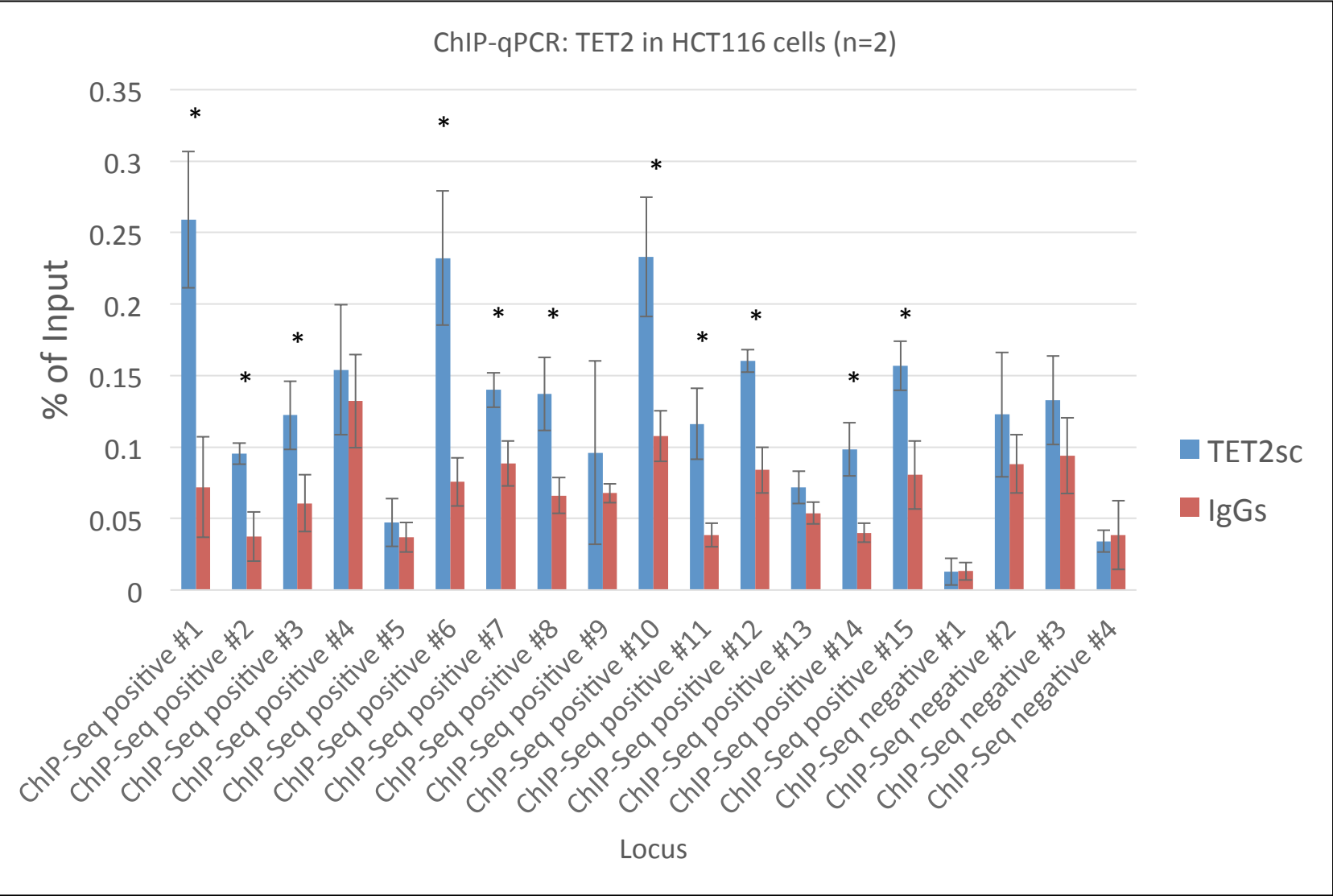

Supplement: Additional file 13: Figure S12. — qPCR Validation of ChIP-seq for TET2 in HCT116 cells. ChIPs using antibodies to TET2 (sc-136926; Santa Cruz) or IgG (sc-136926; Santa Cruz) were performed on the indicated targets (primer sequences are available in Additional file 1). ‘% Input’ represents real-time qPCR values normalised with respect to the input chromatin. Values are represented as means of two independent biological experiments. Asterisks indicate 11 out of 15 loci analysed showed TET2-specific enrichment. [file 13059_2015_605_MOESM13_ESM.pdf]

Uribe-Lewis\_Additional file 14\_Supplementary Fig. 13.

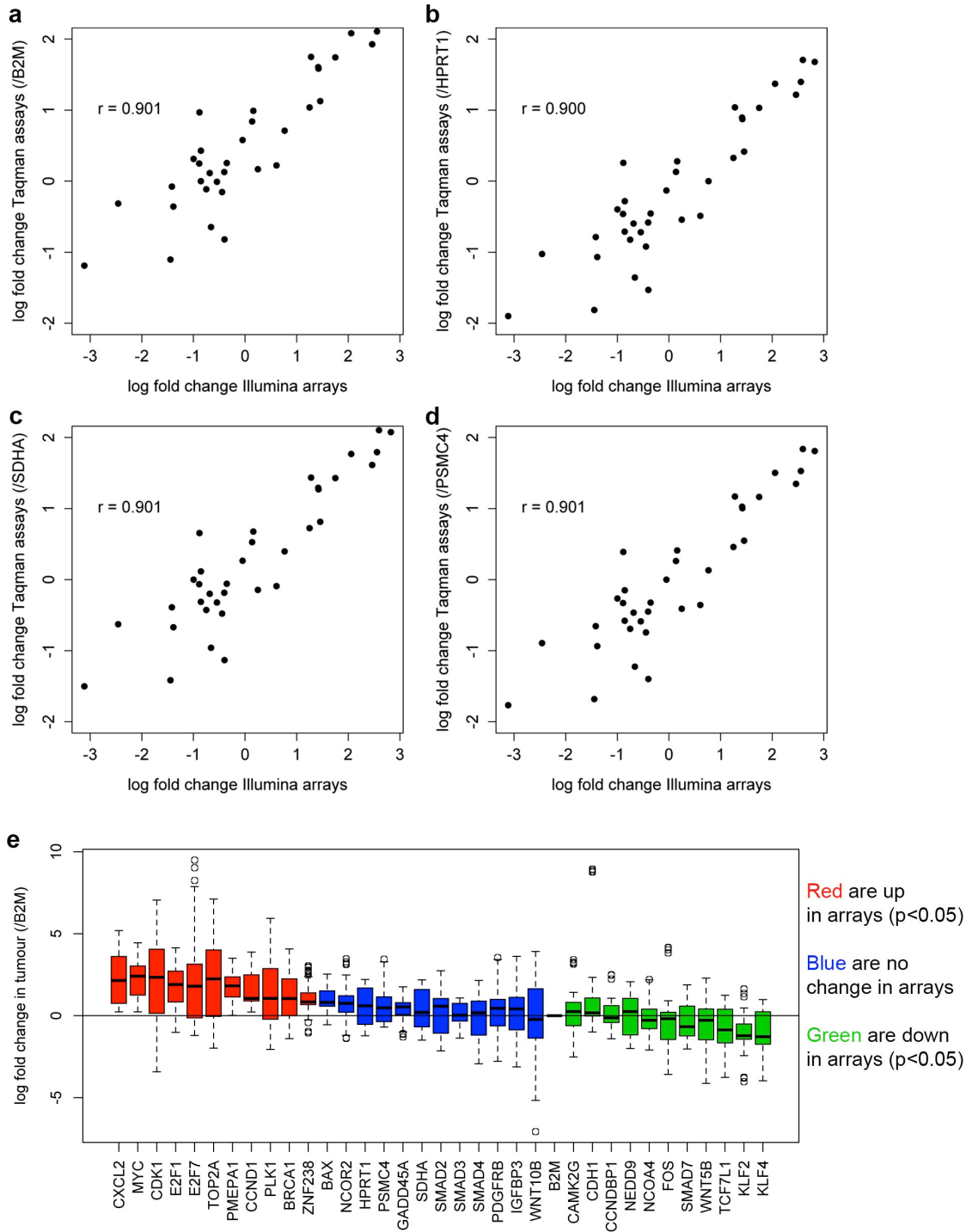

Supplement: Additional file 14: Figure S13. — Validation of the Illumina expression arrays by Taqman qRT-PCR. (a) Plot comparing the fold change in expression from normal to tumour reported by the Illumina arrays and the fold change reported by Taqman qRT-PCR normalised to B2M. Each value from the Illumina arrays used four normals and 14 tumours. Each Taqman value is the average of quadruplicate measurements from 13 normal-tumour pairs. (b to d) As in (a) but normalised to HPRT1, SDHA or PSMC4, respectively. (e) Boxplot of the fold change for the individual assays ordered by their mean. Each assay contains 52 values from quadruplicate measurements of 13 normal-tumour pairs. Taqman assays are listed in Additional file 1. [file 13059_2015_605_MOESM14_ESM.pdf]
