# Supplementary material for: 5-hydroxymethylcytosine marks promoters in colon that resist DNA hypermethylation in cancer
Source: Genome Biol. 2015 Apr 1;16(1):69. doi: 10.1186/s13059-015-0605-5 (PMC4380107; doi:10.1186/s13059-015-0605-5)
Supplement: Additional file 15: — Supplementary information. [file 13059_2015_605_MOESM15_ESM.docx]

**Supplementary information for:**

**5-hydroxymethylcytosine marks promoters in colon that resist DNA hypermethylation in cancer.**

**Santiago Uribe-Lewis^1^, Rory Stark^1^, Thomas Carroll^1^, Mark J. Dunning^1^, Martin Bachman^1^, Yoko Ito^1^, Lovorka Stojic^1^, Silvia Halim^1^, Sarah Vowler^1^, Andy G. Lynch^1^, Benjamin Delatte^2^, Eric J. de Bony^2^, Laurence Colin^2^, Matthieu Defrance^2^, Felix Krueger^3^, Ana-Luisa Silva^4^, Rogier ten Hoopen^4^, Ashraf E.K. Ibrahim^4§^, François Fuks^2§^, Adele Murrell^1,5§*^**

^1^ Cancer Research UK Cambridge Institute, University of Cambridge, Robinson Way, Cambridge CB2 0RE, UK.

^2^ Laboratory of Cancer Epigenetics, Université Libre de Bruxelles, Faculty of Medicine, Route de Lennik 808, 1070 Brussels, Belgium.

^3^ Bioinformatics Group, The Babraham Institute, Babraham Research Campus, Cambridge CB22 3AT, UK.

^4^Department of Pathology Box 231, Level 3, Addenbrooke's Hospital, Hills Road, Cambridge CB2 0QQ, UK.

^5^ Centre for Regenerative Medicine, Department of Biology and Biochemistry, University of Bath, Claverton Down, Bath BA2 7AY, UK.

* Corresponding author: [amm95@bath.ac.uk](mailto:amm95@bath.ac.uk)

**^§^** Joint senior authors.

**Methods.**

***hmeDIP-qPCR:*** pull down as in hmeDIP-seq but using unmodified genomic DNA. Target enrichment was quantified by the standard curve method with a Fast Sybr mix (ABI) and 150nM primers. Amplification and dissociation curves were detected in an ABI HT7900 fast real time system. Primer sequences are listed in additional file 1

***Bisulfite pyrosequencing:*** as previously described [[1](#_ENREF_1)].

***Gluc-MS-qPCR:*** Genomic DNA (50ng) was briefly sonicated then treated with the T4-glucosyltransferase and MspI enzymes following manufacturer’s instructions (Zymo Research). Sybr green qPCR was conducted on a 48.48 Biomark Dynamic Array platform following the manufacturer’s instructions (Fluidigm). Amplicon enrichment, a measure of lack of restriction by MspI and thus presence of 5-hmC, was calculated using the delta Ct method as follows: 2E-((Ct of T4+MspI – Ct of noT4noMspI) - (Ct of noT4+MspI – Ct of noT4noMspI). This value was then normalised to the delta-delta Ct of a loading control locus lacking an MspI site (SFRP2 promoter). See additional file 1 for primer sequences.

***TET mutation screen.*** PCR primer pairs covering TETs coding sequences were designed using ExonPrimer with a 50bp overlap. 10ng genomic DNA were amplified in a 25ul eightplex (four primer pairs) reaction using Phusion polymerase (NEB) in 1x HF buffer and 300nM primers. Twelve reactions per sample were pooled (for a total of 48 amplicons per sample) and normalised to 0.2ng/ul. Illumina libraries were prepared with the Nextera XT kit (Illumina) and quantified by qPCR (Kappa Library Quant kit). 150bp paired end sequencing reads were obtained using a MiSeq Benchtop Sequencer (Illumina) at the CRUK-CI Genomics Core Facility. Aligned bam files (hg19) were processed using indel-realigment, base-quality recalibration and UnifiedGenotyper in The Genome Analysis Toolkit (GATK) v2.4-9-g532efad [[2](#_ENREF_2)] and annotated with annovar [[3](#_ENREF_3)] and SnpEff [[4](#_ENREF_4)]. Base counts at each amplicon were generated using Bioconductor infrastructure [[5](#_ENREF_5)]. Primer sequences are available in additional file 1.

***Western blots.*** Cell lysis of total cell extracts was performed on ice in 50 mM Tris-HCl pH 8, 125 mM NaCl, 1% NP-40, 2 mM EDTA, 1 mM PMSF and protease inhibitory cocktail (Roche) for 25 min. Insoluble material was pelleted by centrifugation at 16000 g for 3 min at 4°C. For cytoplasmic and nuclear extracts preparation the cells were resuspended first in buffer A (10 mM HEPES pH 7.9, 10 mM KCl, 0.1 mM EDTA and 0.1 mM EGTA) supplemented with protease inhibitory cocktail (Roche), 1 mM DTT and 1 mM PMSF. After incubation on ice for 10 min, 0.5% NP-40 was added and the samples vortexed for 5 s. Nuclei were pelleted at 13,200 rpm for 10 s and the cytoplasmic proteins were collected. The pellet was then washed five times with buffer A and resuspended in buffer C (20 mM Hepes pH 7.9, 400 mM NaCl, 1 mM EDTA, 1 mM EGTA, 1 mM DTT, protease inhibitory cocktail (Roche) and 1 mM PMSF). After 10 min on ice, the samples were sonicated and centrifuged at 13,200 rpm for 10 min and nuclear proteins were collected. Protein concentration was determined using the Bradford assay (Bio-Rad). The proteins were denatured, reduced, separated by SDS-PAGE and transferred to nitrocellulose membrane. The membranes were blocked with 5% non-fat dry milk in Tris-buffered saline supplemented with 0.1% Tween-20 (TBST) and incubated with primary antibodies overnight at 4°C. Following three washes with TBST, membranes were incubated with the peroxidase-conjugated secondary antibody, in TBST with 2.5% non-fat dry milk, and immunoreactive proteins were detected using Supersignal West Dura HRP Detection Kit (Thermo-Scientific). Band intensities were quantified using Imagequant (GE Healthcare) and fold change in the knockdown was calculated relative to the shCtrl.

***TET2 ChIP-seq qPCR validation.*** HCT116 cells were crosslinked for 10min at room temperature with 1% formaldehyde then the reaction was quenched with 0.125M glycine and washed twice with 1X cold PBS. ChIP experiments were performed according to the ChIP-IT High Sensitivity kit (Active Motif) protocol. Sonication was performed with a Bioruptor (Diagenode) in cold water with the following settings: 1 cycles of 25 min, sonication strength set on high, with intervals of 30s ON/OFF. 5 ug of rabbit polyclonal antibody for TET2 (sc-136926; Santa Cruz), 1.6 ug of normal rabbit antibody for IgGs (sc-2027; Santa Cruz) were incubated with sheared chromatin overnight at 4°C. After extensive washing steps, ChIPed DNA was eluted, de-crosslinked and purified according to the ChIP-IT High Sensitivity kit (Active Motif) protocol. 3ul of enriched fragmented DNA or 3ul of input, supplemented with 0.5mM of primers and SYBR Green master mix was subjected to 40 cycles of PCR using a LightCycler 480 II (Roche). Percentage of input recovered after immunoprecipitation was calculated using the ΔCt formula: (2^(Ct IP—Ct Input))*100.

***Immunohistochemistry for TET2.*** Tissue or cell line pellet sections were deparaffinised and rehydrated in an ethanol series (100, 95, 70 and 50%). The epitope was retrieved by Proteinase K treatment (1:100 Prot K (Qiagen 19133 in 50mM TRIS pH 7.5, 5min. RT) followed by blocking of endogenous peroxidases, a BSA block (10% BSA in PBS, 30min. in moist chamber) and 3 x 5min washes with PBS-0.05% Tween-20. Primary antibody (TET2 – ab94580) was added in 10% BSA in PBS-0.05% Tween-20 overnight at 4°C in moist chamber and washed off with PBS-0.05% Tween-20. The HRP labeled polymer (DAKO Envision system – 4010) was added for 60min in moist chamber followed by PBS-0.05% Tween-20 washes. DAB plus chromogen for 5min at RT, water wash, hematoxylin stain, de-stain in water, dehydration in an ethanol series (50, 95, 100%) and 100% Xylene. Slides were air dried and mounted with DAKO Glycergel mounting medium.

**References.**

1. Ito Y, Koessler T, Ibrahim AE, Rai S, Vowler SL, Abu-Amero S, Silva AL, Maia AT, Huddleston JE, Uribe-Lewis S, et al: **Somatically acquired hypomethylation of IGF2 in breast and colorectal cancer.** *Hum Mol Genet* 2008, **17:**2633-2643.

2. DePristo MA, Banks E, Poplin R, Garimella KV, Maguire JR, Hartl C, Philippakis AA, del Angel G, Rivas MA, Hanna M, et al: **A framework for variation discovery and genotyping using next-generation DNA sequencing data.** *Nat Genet* 2011, **43:**491-498.

3. Wang K, Li M, Hakonarson H: **ANNOVAR: functional annotation of genetic variants from high-throughput sequencing data.** *Nucleic Acids Res* 2010, **38:**e164.

4. Cingolani P, Platts A, Wang le L, Coon M, Nguyen T, Wang L, Land SJ, Lu X, Ruden DM: **A program for annotating and predicting the effects of single nucleotide polymorphisms, SnpEff: SNPs in the genome of Drosophila melanogaster strain w1118; iso-2; iso-3.** *Fly (Austin)* 2012, **6:**80-92.

5. Lawrence M, Huber W, Pages H, Aboyoun P, Carlson M, Gentleman R, Morgan MT, Carey VJ: **Software for computing and annotating genomic ranges.** *PLoS Comput Biol* 2013, **9:**e1003118.

6. Ye T, Krebs AR, Choukrallah MA, Keime C, Plewniak F, Davidson I, Tora L: **seqMINER: an integrated ChIP-seq data interpretation platform.** *Nucleic Acids Res* 2011, **39:**e35.

7. Bock C, Tomazou EM, Brinkman AB, Muller F, Simmer F, Gu H, Jager N, Gnirke A, Stunnenberg HG, Meissner A: **Quantitative comparison of genome-wide DNA methylation mapping technologies.** *Nat Biotechnol* 2010, **28:**1106-1114.

8. Hansen KD, Timp W, Bravo HC, Sabunciyan S, Langmead B, McDonald OG, Wen B, Wu H, Liu Y, Diep D, et al: **Increased methylation variation in epigenetic domains across cancer types.** *Nat Genet* 2011, **43:**768-775.
